# Investigating the microbial and metalloprotease sequestration properties of superabsorbent wound dressings

**DOI:** 10.1038/s41598-022-08361-3

**Published:** 2022-03-19

**Authors:** Gurdeep Singh, Charlotte Byrne, Helen Thomason, Andrew J. McBain

**Affiliations:** 1 Division of Pharmacy and Optometry, Stopford Building, Faculty of Biology, Medicine and Health, School of Health Sciences, The University of Manchester, Oxford Road, Manchester, M13 9PT UK; 2 Medical Solutions Division, 3M, King Edward Court, King Edward Road, Knutsford, WA16 0BE UK

**Keywords:** Biochemistry, Biological techniques, Microbiology

## Abstract

Exudate production is a natural part of the wound healing process, however levels of exudate need to be appropriately managed to maintain a moist wound environment which supports healing. An overly-exuding wound creates an environment favourable to bacterial growth. In recent years, a significant increase in commercially available superabsorbent dressings have become available which claim to absorb and retain excess exudate and its components. However, the effectiveness of these dressings in sequestering and retaining bacteria and host-derived proteins has not been compared. We have therefore investigated several superabsorbent dressings for their ability to absorb and retain bacteria (*Staphylococcus aureus* and *Pseudomonas aeruginosa*), their impact on bacterial viability, and their ability to sequester matrix metalloproteinases (MMP)-2 and 9 over 7 days. Whilst all dressings could sequester bacteria, some dressings internalised bacteria more effectively. There was considerable variation in bacterial viability within the dressings’ core, as well as differences in bacterial retention. Some dressings effectively internalised and retained bacteria over time, whereas other dressings retained significantly less. These differences were reflected visually using scanning electron microscopy. Most dressings fully sequestered MMP-2 and 9. These data illustrate differences in the ability of superabsorbent dressings to absorb and retain exudate and its components.

## Introduction

Chronic wounds are a major cause of morbidity and social isolation, estimated to affect over 600 million people globally in 2015^1,2^. They are also a global financial burden, costing healthcare services in the US an estimated $20 billion^3,4^ and the UK an estimated £8.3 billion from 2017–2018^5^. These figures are likely to increase with an ageing population, where life expectancy in developed countries has increased considerably^6^. If the wound fails to heal promptly, the risk of bacterial infection and the formation of community structures such as biofilms increases^7^. Notably, these wounds can produce various levels of exudate.

Exudate is important for creating a moist wound environment and promoting healing^8,9^. In acute wounds, exudate contains white blood cells to control microorganisms, nutrients and growth factors to promote cell proliferation and migration, and proteases to remodel the damaged extracellular matrix^10^. As an acute wound heals, the concentration of exudate diminishes. In contrast, many chronic wounds exhibit excess exudate production, and the exudate composition differs from that of an acute wound, with elevated white blood cells and proteases^11,12^. This overproduction of exudate can cause tissue damage and increases the risk of infection, which is one mechanism that leads to impaired healing^13^. A further consideration is that saturated dressings or those that have poor retention properties, will expose the skin to excess moisture. Although a moist environment is important for healing^14,15^, an excess can cause damage^16^. The management of excess exudate with an appropriate dressing is therefore beneficial to wound healing. Therefore, to combat high levels of exudate, such wounds may be managed with dressings containing ‘superabsorbent polymers’, designed to absorb high volumes of exudate and retain this exudate under pressure. These superabsorbent dressings are a relatively new type of dressing which are becoming an increasingly popular method of managing moderate to highly exuding wounds.

The moist, warm wound environment provides favourable conditions for microbial growth, which may initiate an infection if unmanaged^17^. When covered with a superabsorbent dressing, bacteria may be internalised within the inner core of the dressing. However, few studies have detailed the fate of the bacteria internalised within the dressing. For instance, internalised microorganisms could still be viable within the dressing and therefore capable of causing harm if the dressing is left unchanged. Furthermore, wound bacteria can form biofilms that are highly tolerant to conventional treatment^18,19^.

Another aspect of delayed healing involves the dysregulated recruitment of healing factors such as matrix metalloproteinases (MMPs). MMPs comprise a family of proteins that are part of the normal wound healing process, of which MMP-2 and 9 are reported to be the most important^20–22^, where they function to remodel the extracellular matrix, promote cell migration, and re-epithelisation^23^. MMPs have been found in high levels in the exudate of chronic wounds^17^. Managing wound exudate is therefore crucial in promoting timely wound closure. Previous work has shown that superabsorbent dressings can absorb MMPs^20^, as well as other proteins involved in the healing process such as PMN-elastase^24,25^. However, comparative studies between different superabsorbent dressings in this context are rare^25^.

Given the potential benefits of superabsorbent dressings, there has been a large increase in the number of commercially available dressings that claim to effectively manage wound exudate and its components. We have therefore investigated a range of commercially available superabsorbent dressings, exploring the ability of these dressings to sequester and retain wound pathogens, *Staphylococcus aureus* and *Pseudomonas aeruginosa.* We assessed the viability and burden of bacteria within the inner core of the dressings over time, the localisation of bacteria on the outer wound contact layer and the ability to sequester and retain MMPs.

## Materials and methods

### Choice of dressings

Commercially available dressings were chosen for this study. Dressings tested included: ConvaMax™ Superabsorber (ConvaTec Ltd, Deeside, UK), 3M™ Kerramax™ Care Super-Absorbent Dressing (3M, Knutsford, UK), Kliniderm® Superabsorbent (H&R Healthcare Ltd., Hull, UK), Mextra® Superabsorbent (Mölnlycke® Health Care Limited, Milton Keynes, UK), Vliwasorb® Pro (L&R Medical UK Ltd., Burton on Trent, UK), and Zetuvit® Plus (Paul Hartmann Ltd., Heywood, Manchester, UK). A table summarising dressing dimensions **(**Table 1**)** is included below:

**Table 1:** Summary of superabsorbent dressing specifications.

Dressing	Outer dimensions (L × W, cm)	Inner core dimensions (L × W, cm)*
ConvaMax™ Superabsorber	10 × 10	7.5 × 7.5
3M™ Kerramax™ Care Super-absorbent Dressing	10 × 10	8.4 × 6.7
Kliniderm® Superabsorbent	10 × 10	7 × 7
Mextra® Superabsorbent	12.5 × 12.5	9.8 × 9.6
Vliwasorb® Pro	12.5 × 12.5	8.5 × 8.6
Zetuvit® Plus	10 × 10	9.8 × 10.7

*As determined by investigator.

### Free swell and fluid retention test

A solution of deionised water (diH_2_O), containing 143 mM NaCl and 3 mM CaCl_2_ was prepared. Each dressing was weighed, before being placed into an excess of solution in a sealed container and into an incubator at 37 °C. After 30 min of incubation, the dressing was removed and suspended at the corner with sterile forceps for 30 s to allow fluid release. The dressing was subsequently re-weighed. Following this test, the dressing was placed onto a perforated surface and weights applied to the dressing, to provide either 30 mmHg or 40 mmHg of moderate pressure^26^ relative to the pad size. Specifically, pressure was converted from mmHg to gfcm^−1^ and then weight was applied as required for the calculated superabsorbent pad size. This was applied evenly to the pad by using a plastic coaster of a size similar to that of the pad to more evenly disperse the pressure over it. After 30 s of pressure, the dressing was re-weighed. As these dressings are not available in a single standard size, the total fluid absorbed by the dressings was measured and adjusted to reflect the volume of fluid absorbed per 100 cm^2^ of dressing. The absorbency was then calculated based on two separate measurements; firstly, based on the size of the superabsorbent core, and secondly, based on the overall size of the dressing. The superabsorbent core of the dressing is smaller than the overall size of the dressing to allow the core to swell during fluid uptake without the dressing splitting open. This measurement permits a direct comparison of the fluid handling properties of the superabsorbent core.

### Bacterial transfer from dressings

Dressings were placed into 120 mm square Petri dishes (Scientific Laboratory Supplies Ltd., Nottingham, UK) and 10 ml of bacterial inoculum containing between 10^6^–10^7^ CFU/ml bacteria in Mueller–Hinton Broth (Sigma-Aldrich Company Ltd., Kent. UK), was placed under each dressing daily. Two bacteria were tested, either *Pseudomonas aeruginosa* (NCTC 10781) or *Staphylococcus aureus* (NCTC 6571). Dressings were removed after 1, 3 and 7 days of inoculation and transferred to Mueller–Hinton agar (Sigma-Aldrich Company Ltd). These timepoints were chosen to simulate when dressings may be changed according to different clinical practices. Plates were incubated at 37 °C overnight for 24 h. Dressings were removed and plates were incubated at 37 °C for a further 24 h. Bacterial growth was imaged using a Nikon camera (Nikon UK Limited, Kingston, UK) with the following settings: ISO 100, Raw + Fine, aperture F11, matrix metering and autofocus. Plates were macroscopically assessed for differences in the transfer of bacteria to agar, between dressings.

### Bacterial release from dressings

Dressings were inoculated as described above. However, at each time point, dressings were removed and an 8 mm biopsy (Scientific Laboratory Supplies Ltd.) was obtained from the inner core as centrally as possible. This biopsy was transferred into 3 ml of Dey Engley Neutralising Broth (Sigma-Aldrich Company Ltd.) containing 2 mm borosilicate solid-glass beads for lysing (Sigma-Aldrich Company Ltd.). Dressing biopsies were vortexed at full speed for 3 min. Serial dilutions were performed of each lysate and CFU/ml determined by plating and performing colony counts.

### Bacterial localisation within dressings

At each time point, a 5 mm piece of the outer membrane of each dressing was obtained as centrally as possible. These pieces were fixed in 2.5% glutaraldehyde and 4% freshly prepared formaldehyde in 0.1 M HEPES buffer; for 2 h. The samples were then dehydrated in a graded (30–100%) ethanol series for 1 h and critical point-dried in CO_2_ using a Quorum K 850 (Quorum Technologies Limited, Lewes, UK). Finally, the samples were sputter-coated with gold–palladium alloy using a Quorum SC7620 (Quorum Technologies Ltd.) and observed with an FEI Quanta 250 scanning electron microscope (ThermoFisher Scientific, Paisley, UK).

### Bacterial viability

Bacterial viability was investigated via flow cytometry using the BacLight Bacterial Viability Kit for Microscopy (ThermoFisher Scientific). Dressings were inoculated as described above and each 8 mm biopsy was lysed with glass beads in 10 ml of sterile diH2O. 2 ml of this solution was filtered through a 0.40 µm sterile filter and stained with 6 µl of prepared BacLight reagent. From each dressing lysate, 10,000 cells were acquired using a BD LSR Fortessa X-20 (BD Biosciences, San Jose, CA) and data have been reported as percentage proportions of live and dead bacteria out of the 10,000 bacteria sampled. Data were analysed using FlowJo V10.6.1 software (Tree Star, Oregon, USA) (https://www.flowjo.com/). The gating strategy is shown in Supplementary Fig. 1.

### Sequestration of MMPs

Solutions (6 ng/ml) were reconstituted from Recombinant Human MMP-2 (R&D Systems, Abingdon, UK) and Recombinant Human MMP-9 (R&D Systems) in the following buffers: MMP-2 (20 mM Tris–HCL, 150 mM NaCl, 5 mM CaCl_2_ and 0.05% (w/v) Brij­35, pH 7.4) and MMP-9 (50 mM Tris–HCL, 150 mM NaCl, 10 mM CaCl_2_ and 0.05% (w/v) Brij-35, pH 7.5). A 5 mm piece of each dressing was soaked with 1 ml of each solution and incubated at 37 °C at 105 rpm for 4 days. After this incubation, supernatants were collected and ELISAs were performed according to the manufacturer’s instructions (Quantikine ELISA Total MMP-2 and Quantikine ELISA Human MMP-9—all R&D Systems) were imaged using a VersaMax microplate reader (Molecular Devices, San Jose, CA).

### Statistical analysis

All statistical analysis was performed using GraphPad Prism V8 (GraphPad Software, La Jolla, CA) (https://www.graphpad.com/). Two analysis of variance (ANOVA) with a Tukey’s post hoc test was used to compare fluid absorbency and retention between dressings. Additionally, Two way ANOVA with a Tukey’s post hoc test was used to compare bacterial release over time and bacterial viability between dressings. One way ANOVA was used to compare the percentage absorption of MMPs between dressings.

## Results

### Comparable retention of fluid between superabsorbent dressings

**Figure 1:**
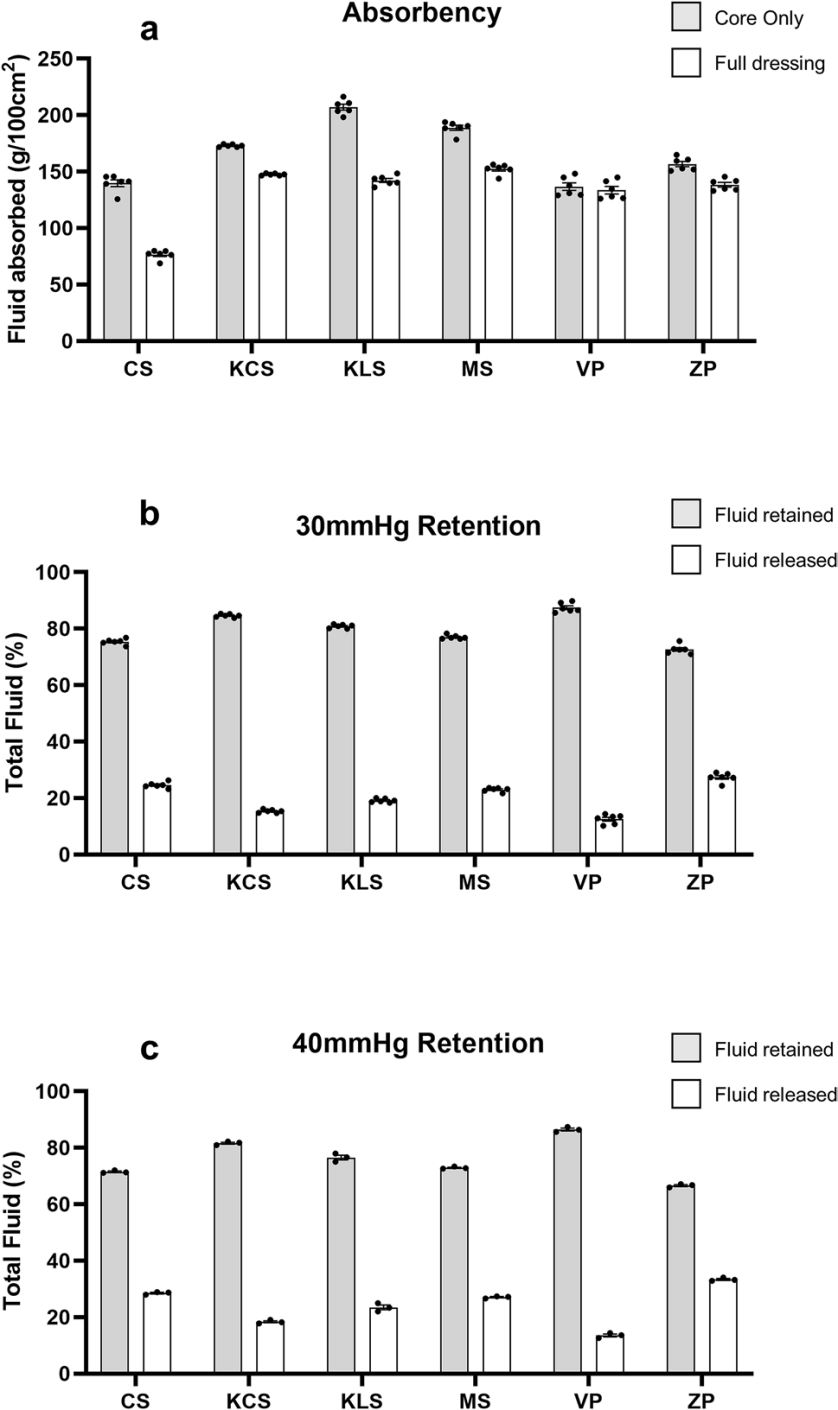
Absorbency and retention of fluid by superabsorbent dressings. Superabsorbent wound dressings were placed into an excess of salt solution. Dressings were weighed after 30 min of soaking at 37 °C and data plotted as g/100 cm^2^ (**a**), based on either the size of the inner core or the whole area of the dressing. Pressure, either 30 mmHg (**b**) or 40 mmHg (**c**) relative to pad size, was then applied to each dressing and re-weighed. Data are shown as percentage fluid retained and released. The following dressings were tested: ConvaMax™ Superabsorber (CS), 3M Kerramax Care Super-Absorbent Dressing (KCS), Kliniderm Superabsorbent (KLS), Mextra Superabsorbent (MS), Vliwasorb Pro (VP) and Zetuvit Plus (ZP). Figure produced in GraphPad Prism V8.

Superabsorbent dressings are designed to manage high volumes of wound exudate. We therefore tested the ability of six commercially available superabsorbent dressings to absorb fluid (Fig. 1a) and retain fluid under two different pressures (Fig. 1b,c). Given the number of comparisons, significant differences are illustrated in Supplementary Tables 1–3. All dressings absorbed a large volume of fluid (Fig. 1a). Kliniderm Superabsorbent had the largest volume of fluid absorbed when considering core size (mean 207 g/100 cm^2^), significantly higher than all other dressings. This was followed by Mextra Superabsorbent (188 g/100 cm^2^), Kerramax Care Dressing (173 g/100 cm^2^), Zetuvit Plus (156 g/100 cm^2^), ConvaMax Superabsorber (139 g/100 cm^2^) and Vliwasorb Pro (136 g/100 cm^2^). When the full size of the dressing was considered, fluid absorption was generally similar between all dressings, except for ConvaMax Superabsorber (76 g/100 cm^2^) which was significantly lower than all the other dressings (Supplementary Table 1).

There was variability between dressings with respect to fluid retention. At 30 mmHg pressure (Fig. 1b) Vliwasorb Pro retained significantly more fluid (mean 87%) than all other dressings. This was followed by Kerramax Care Dressing (85%), Kliniderm Superabsorbent (81%), Mextra Superabsorbent (77%), ConvaMax Superabsorber (75%) and Zetuvit Plus (73%). A similar trend was seen when when 40 mmHg was applied (Fig. 1c).

### Variable transfer of bacteria to agar between dressings

**Figure 2:**
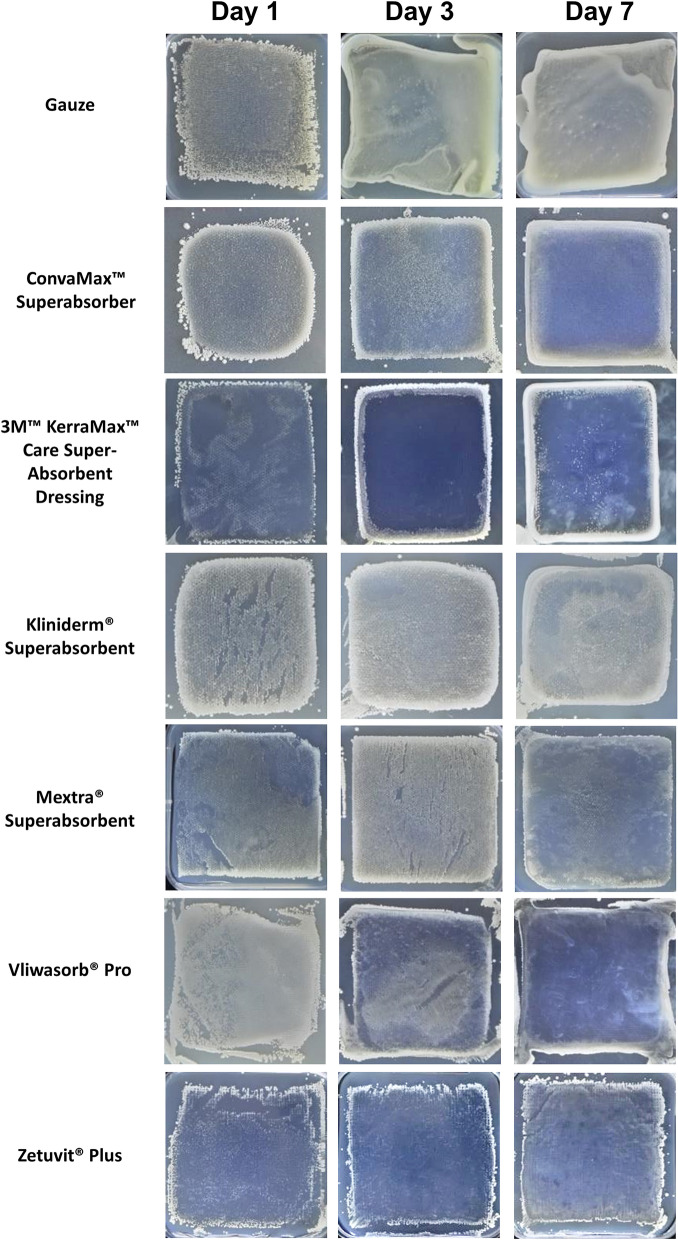
Bacteria transferred to surfaces by superabsorbent dressings. Superabsorbent wound dressings were inoculated with ~ 1 * 10^7^ CFU/ml of *Staphylococcus aureus* daily, over 7 days in triplicate. On 1, 3 and 7 days of inoculation, dressings were transferred to agar and cultured overnight at 37 °C. Dressings were removed and cultured overnight at 37 °C. Plates were imaged for bacterial growth. Representative images for each dressing are displayed. Figure produced in Microsoft PowerPoint 365.

**Figure 3:**
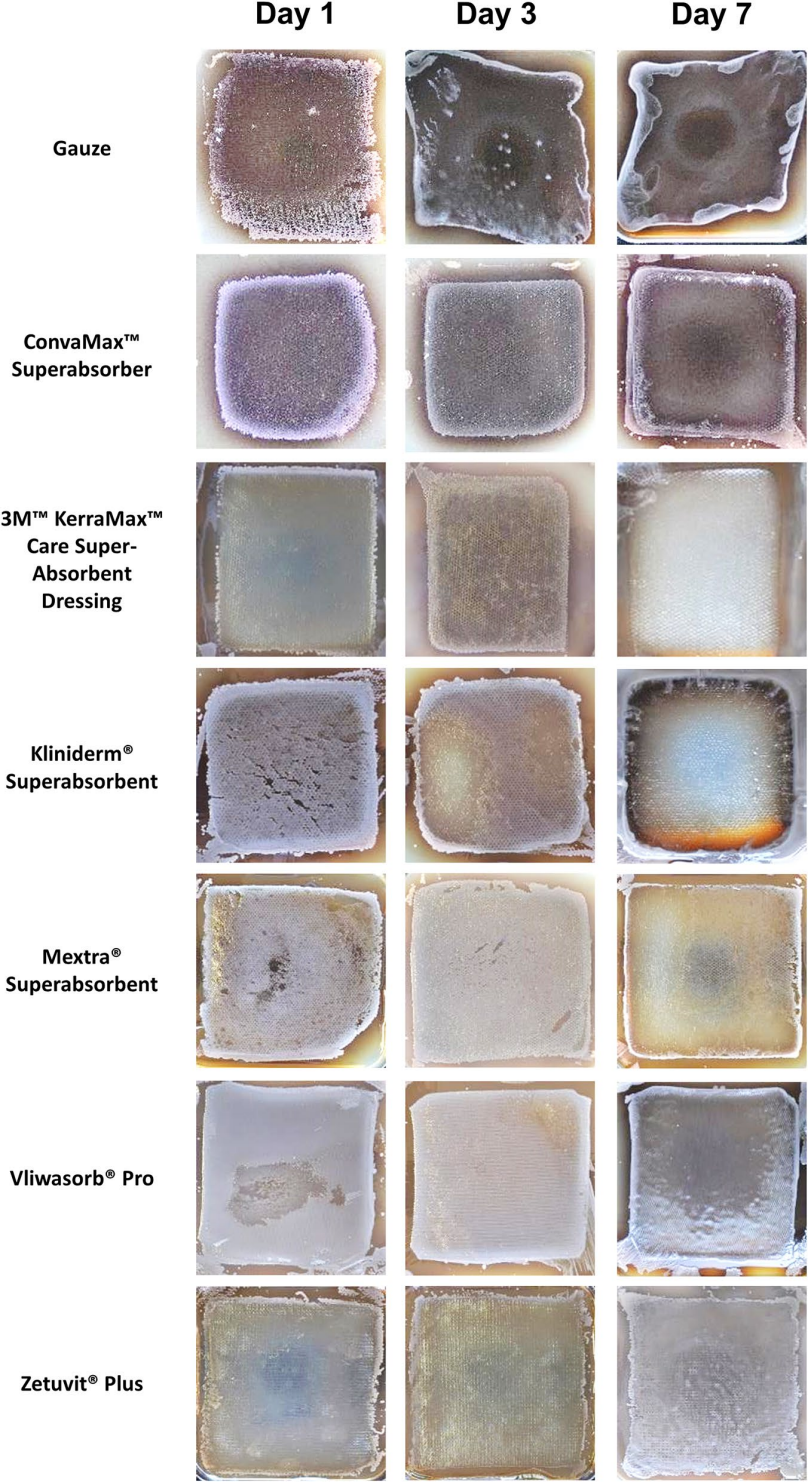
Bacteria transferred to surfaces by superabsorbent dressings. Superabsorbent wound dressings were inoculated with ~ 1 * 10^7^ CFU/ml of *Pseudomonas aeruginosa* daily, over 7 days in triplicate. On 1, 3 and 7 days of inoculation, dressings were transferred to agar and cultured overnight at 37 °C. Dressings were removed and cultured overnight at 37 °C. Plates were imaged for bacterial growth. Representative images for each dressing are displayed. Figure produced in Microsoft PowerPoint 365.

We investigated how easily bacteria can be transferred from a dressing to a surface, at three key time points. There was a great deal of variability in the transfer of bacteria to agar, both between the dressings and the bacteria themselves (Figs. 2, 3), that could be observed macroscopically.

When inoculating superabsorbent dressings with *S. aureus* (Fig. 2), the gauze control did not retain the bacteria, with opaque sheets of *S. aureus* forming at each time point on the agar plates. Two superabsorbent dressings had clear regions around the inner core at each time point, Kerramax Care Dressing and Zetuvit Plus, indicating the bacteria were locked within the dressings’ inner core throughout the 7 days tested. Notably, Vliwasorb Pro, ConvaMax Superabsorber and to a lesser extent, Mextra Superabsorbent, released bacteria at the early time-points but performed better at later time points. Specifically, Vliwasorb Pro and ConvaMax Superabsorber had visually clearer regions around the inner core on day 7, compared to day 1. Mextra Superabsorbent visually transferred a large number of bacteria on day 3, but less on day 7.

The trends for *P. aeruginosa* transfer to agar were less clear macroscopically (Fig. 3). Although sheets of *P. aeruginosa* formed with gauze, the growth appeared patchy. It was observed that Zetuvit Plus transferred few bacteria to agar on day 1 and 3, but transferred a great deal more on day 7. ConvaMax Superabsorber, Kliniderm Superabsorbent and Mextra Superabsorbent had clear zones at day 7 but performed less well at earlier timepoints. Vliwasorb Pro showed growth at all timepoints. Kerramax Care Dressing appeared to have comparatively clearer regions on the agar, devoid of bacterial growth, compared to the other dressings at all time points indicating that the bacteria remained within the dressings inner core.

### Bacterial release differs between dressings

**Figure 4:**
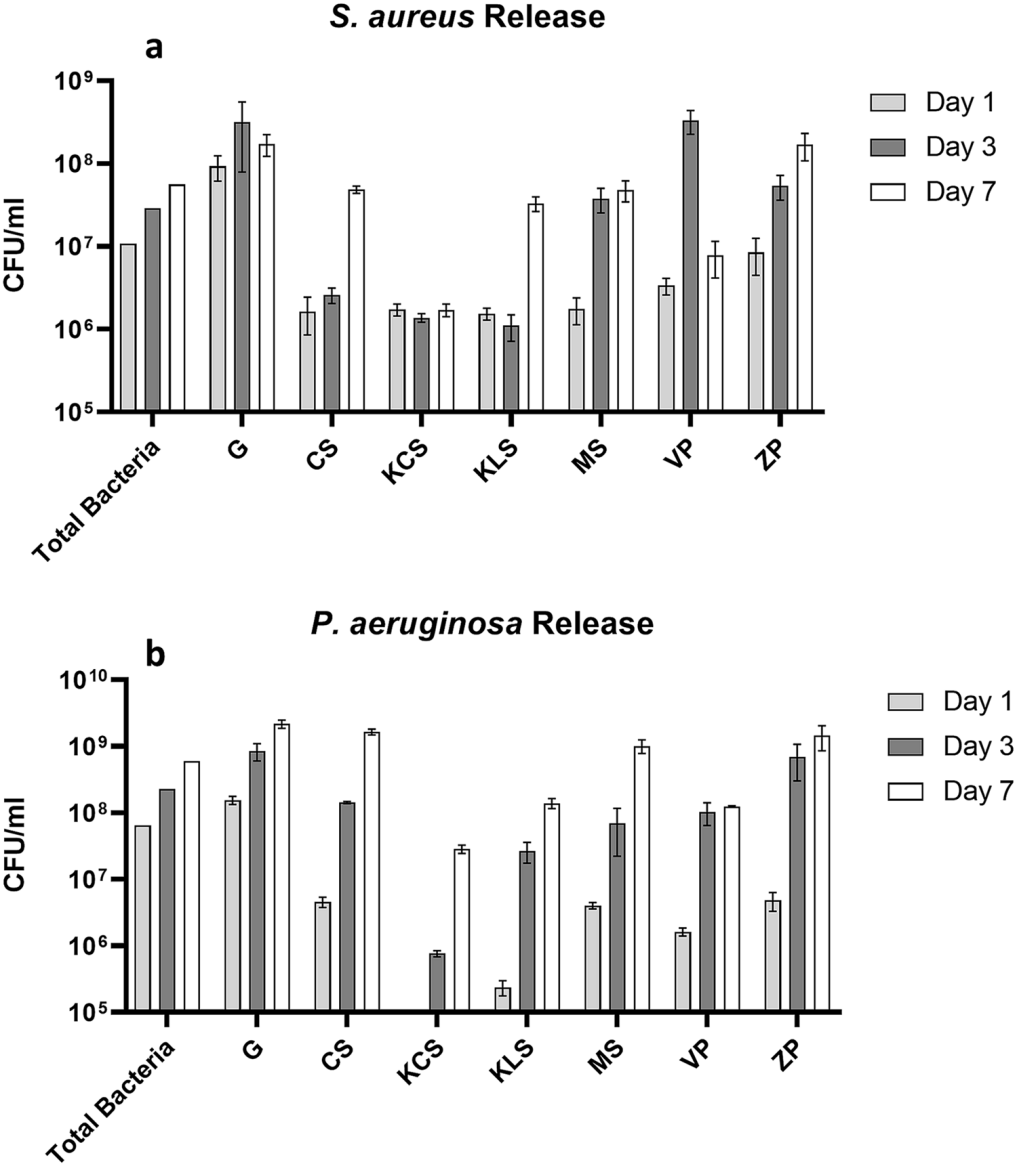
Variable bacterial release of superabsorbent dressings. Superabsorbent wound dressings were inoculated daily with 1 * 10^7^ CFU/ml of either *Staphylococcus aureus* or *Pseudomonas. aeruginosa* daily, over 7 days in triplicate. On 1, 3 and 7 days of inoculation, dressings were lysed, any antimicrobial activity neutralised and bacterial load was quantified via serial dilutions. Colony-forming units (CFU/ml) determined via colony counts are shown for *S. aureus* (**a**) and *P. aeruginosa* (**b**). The following dressings were tested: Gauze (G), ConvaMax Superabsorber (CS), 3M Kerramax Care Super-Absorbent Dressing (KCS), Kliniderm Superabsorbent (KLS), Mextra Superabsorbent (MS), Vliwasorb Pro (VP) and Zetuvit Plus (ZP). Data are shown as mean (+/− SEM, n = 3). Figure produced in GraphPad Prism V8.

We quantified how much bacteria can be physically released by the dressings, as this could increase the risk of infection developing within the wound. We have included the total bacteria applied to the dressings at each point, to further illustrate the effectiveness of each dressing at retaining bacteria. The CFU/ml of each dressing has been compared against the total bacteria that was applied to it, at each time point, With respect to *S. aureus* (Fig. 4a), gauze had the highest bacterial burden at each time point, releasing enough bacteria to correspond with the total bacteria applied. ConvaMax Superabsorber released tenfold lower bacteria than the total bacteria applied at each time point. Kerramax Care released the fewest bacteria of all the dressings tested at all timepoints, releasing tenfold lower bacteria than the total applied at day 1 and 100-fold lower than the total bacteria applied on day 3 and 7. Kliniderm Superabsorbent followed a similar trend at day 1 and 3, but released tenfold more bacteria than Kerramax Care Dressing on day 7. Vliwasorb Pro released tenfold less bacteria than the total applied on day 1, but this rose to be comparable to the total applied at day 3, before unexpectedly dropping to 100-fold lower on day 7. ConvaMax Superabsorber, Mextra Superabsorbent and Zetuvit Plus all released tenfold less bacteria than the total applied on day 1. ConvaMax Superabsorber released 100-fold less at day 3 and tenfold less on day 7. However, Mextra Superabsorbent and Zetuvit Plus released tenfold less bacteria on day 3 and day 7. Significant differences between the dressings are illustrated in Supplementary Table 4.

With respect to *P. aeruginosa* (Fig. 4b), gauze had the highest bacterial concentration across all timepoints. As with *S. aureus* Kerramax Care Dressing had the greatest bacterial retention, exhibiting no detectable bacteria on day 1, and had 1000-fold fewer bacteria on day 3 and tenfold fewer bacteria on day 7, when compared to the total bacteria applied. Kliniderm Superabsorbent had 100-fold less bacteria on day 1, tenfold less on day 3 and was comparable to total bacteria applied at day 7. ConvaMax Superabsorber, Vliwasorb Pro and Zetuvit Plus followed the same trend, with tenfold fewer bacteria at day 1 and were comparable to total bacteria applied on day 3 and day 7. Mextra Superabsorbent had tenfold fewer bacteria at day 1 and 3, before rising to be comparable with total bacteria applied on day 7. Significant differences between the dressings are illustrated in Supplementary Table 5.

### Bacterial viability

**Figure 5:**
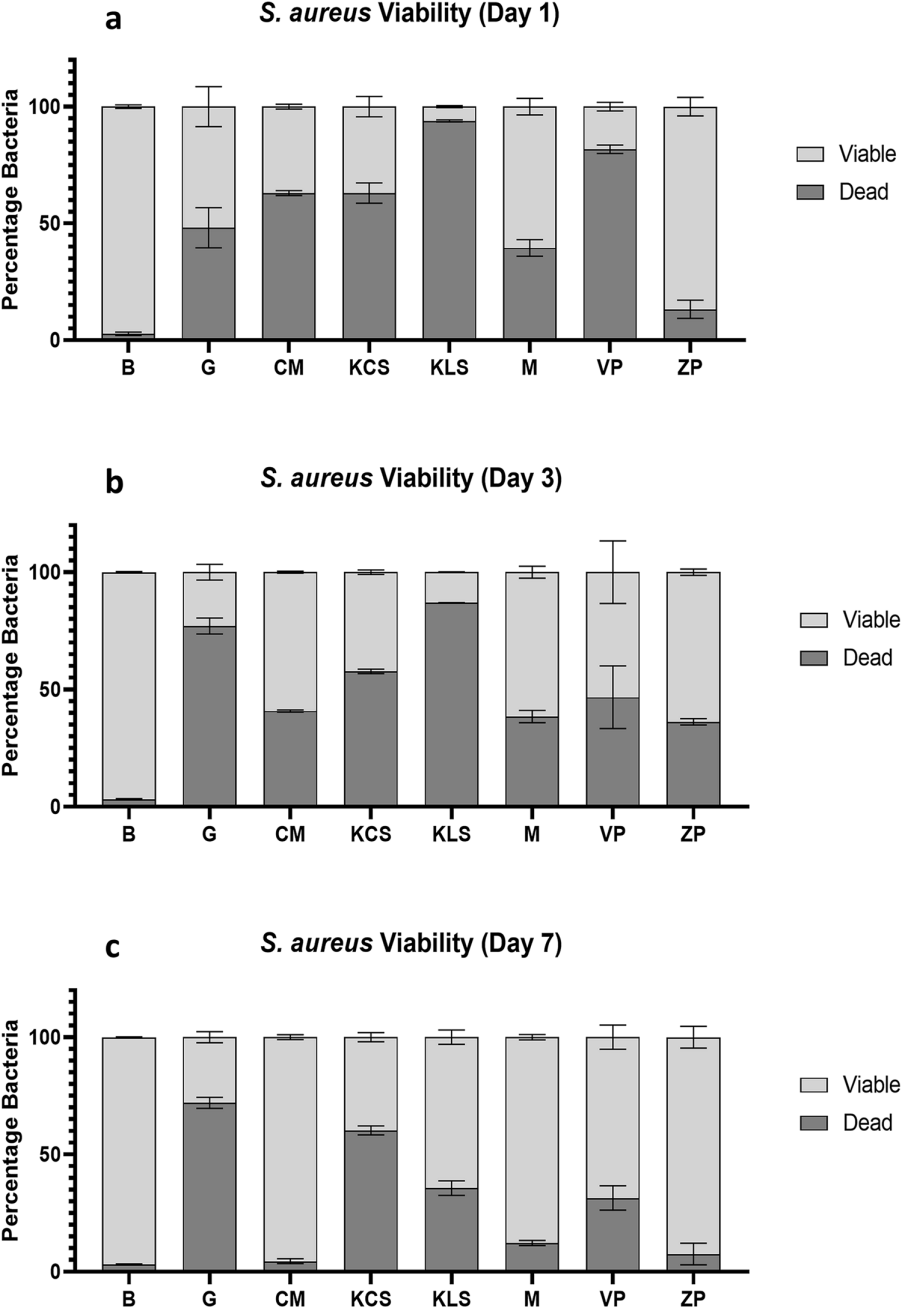
Viability of *Staphylococcus aureus* in superabsorbent dressings. Superabsorbent wound dressings were inoculated with 1 * 10^7^ CFU/ml of *Staphylococcus aureus*, over 7 days in triplicate. On 1 (**a**), 3 (**b**) and 7 (**c**) days of inoculation, dressings were lysed and stained with a bacterial viability kit, to determine viability via flow cytometry. The following dressings were tested: Gauze (G), ConvaMax Superabsorber (CS), 3M Kerramax Care Super-Absorbent Dressing (KCS), Kliniderm Superabsorbent (KLS), Mextra Superabsorbent (MS), Vliwasorb Pro (VP) and Zetuvit Plus (ZP). A broth control was included (B). Data are shown as mean (+ /− SEM). Figure produced in GraphPad Prism V8 and data were analysed using FlowJo V10.6.1.

**Figure 6:**
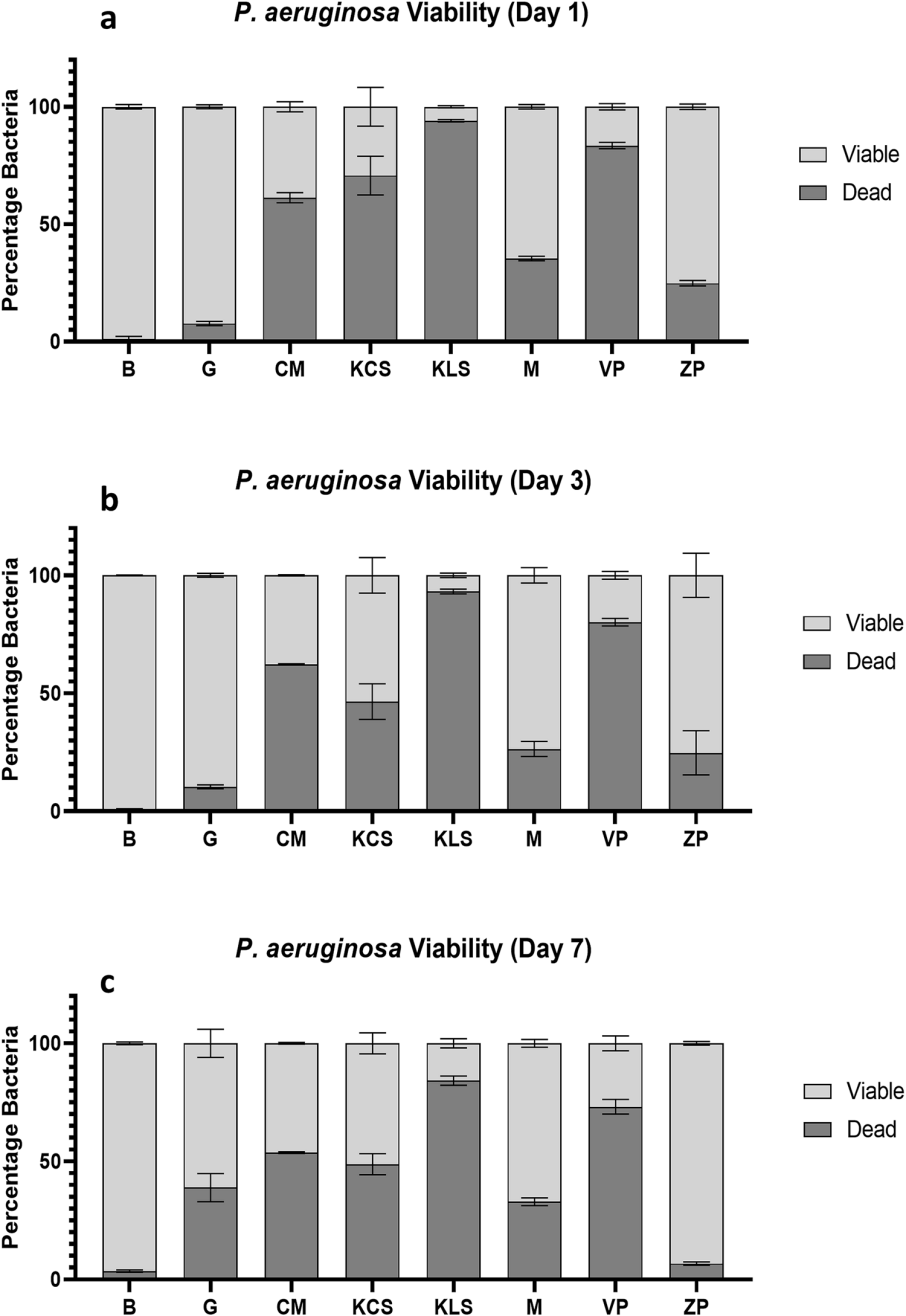
Viability of *Pseudomonas aeruginosa* in superabsorbent dressings. Superabsorbent wound dressings were inoculated daily with 1 * 10^7^ CFU/ml of *Pseudomonas aeruginosa*, over 7 days in triplicate. On 1 (**a**), 3 (**b**) and 7 (**c**) days of inoculation, dressings were lysed and stained with a bacterial viability kit, to determine viability via flow cytometry. The following dressings were tested: Gauze (G), ConvaMax Superabsorber (CS), 3M Kerramax Care Super-Absorbent Dressing (KCS), Kliniderm Superabsorbent (KLS), Mextra Superabsorbent (MS), Vliwasorb Pro (VP) and Zetuvit Plus (ZP). A broth control was included (B). Data are shown as mean (+ /− SEM). Figure produced in GraphPad Prism V8 and data were analysed using FlowJo V10.6.1.

Understanding whether the bacteria internalised within the dressings are alive or dead is particularly important, as dressings containing viable bacteria would be hazardous to patients and clinicians, particularly if the dressings’ retentive properties were poor. We, therefore, investigated the viability of bacteria bound to the superabsorbent dressings core using flow cytometry (Figs. 5, 6). Inoculated broth was used as an additional control. Given the number of statistical comparisons for these data, significant differences are illustrated in Supplementary Tables 6–11, for each time point and bacterium.

With respect to *S. aureus* (Fig. 5), on day 1 (Fig. 5a), almost all the bacteria in broth were viable (97%). Gauze, Mextra Superabsorbent and Zetuvit Plus had the most viable bacteria at this time point, with an average of 51%, 60% and 86% viable bacteria respectively. Kerramax Care Dressing and ConvaMax Superabsorber functioned comparably on day 1, with similar proportions of viable bacteria (an average of 37% for both dressings). Vliwasorb Pro released an average of 18% viable bacteria, with Kliniderm Superabsorbent releasing the smallest proportion of viable bacteria of the dressings (3%).

On day 3 (Fig. 5b), bacteria in the control broth (no dressing) remained viable (96%). Of the dressings, Zetuvit Plus, Mextra Superabsorbent, and ConvaMax Superabsorber performed comparably, with 63%, 61% and 59% of viable bacteria respectively. This was followed by Vliwasorb Pro (53% viable bacteria) and Kerramax Care Dressing (42% viable bacteria). Gauze and Kliniderm Superabsorbent released the fewest viable bacteria at this time point, with 22% and 13% respectively.

On day 7 (Fig. 5c), broth maintained bacterial viability (96%). ConvaMax Superabsorber, Zetuvit Plus and Mextra Superabsorbent performed comparably again (95%, 92% and 87% viable bacteria respectively). This was followed by Vliwasorb Pro (68%) and Kliniderm Superabsorbent (64%). Kerramax Care Dressing released the fewest viable bacteria of the superabsorbents tested at this time point (39%). Surprisingly, gauze released the fewest viable bacteria overall (27%).

Concerning *P. aeruginosa* (Fig. 6), at day 1 (Fig. 6a), nearly all of the bacteria were viable in broth (98%). Gauze released the highest amount of viable bacteria (92%). Of the superabsorbent dressings, Zetuvit Plus released the most viable bacteria (75%), followed by Mextra Superabsorbent (64%). ConvaMax Superabsorber released 38% viable bacteria, whereas Kerramax Care released 29%. Vliwasorb Pro and Kliniderm Superabsorbent released the fewest viable bacteria (16% and 5% respectively).

On day 3 (Fig. 6b), the bacteria within broth were almost entirely viable (98%). Gauze released the highest amount of viable bacteria (89%). Zetuvit Plus and Mextra Superabsorbent performed comparably (75% and 73% respectively). This was followed by Kerramax Care Dressing (53%) and ConvaMax Superabsorber (37%). Vliwasorb Pro (19%) and Kliniderm Superabsorbent (6%) released the fewest viable bacteria.

On day 7 (Fig. 6c), broth maintained bacterial viability (96%). Zetuvit Plus released the highest proportion of viable bacteria (93%), followed by Mextra (67%) and gauze (61%). Kerramax Care and ConvaMax Superabsorber released 51% and 46% viable bacteria respectively. Vliwasorb Pro and Kliniderm Superabsorbent once again released the fewest viable bacteria (26% and 15%).

### Variable bacterial localisation in dressings

**Figure 7:**
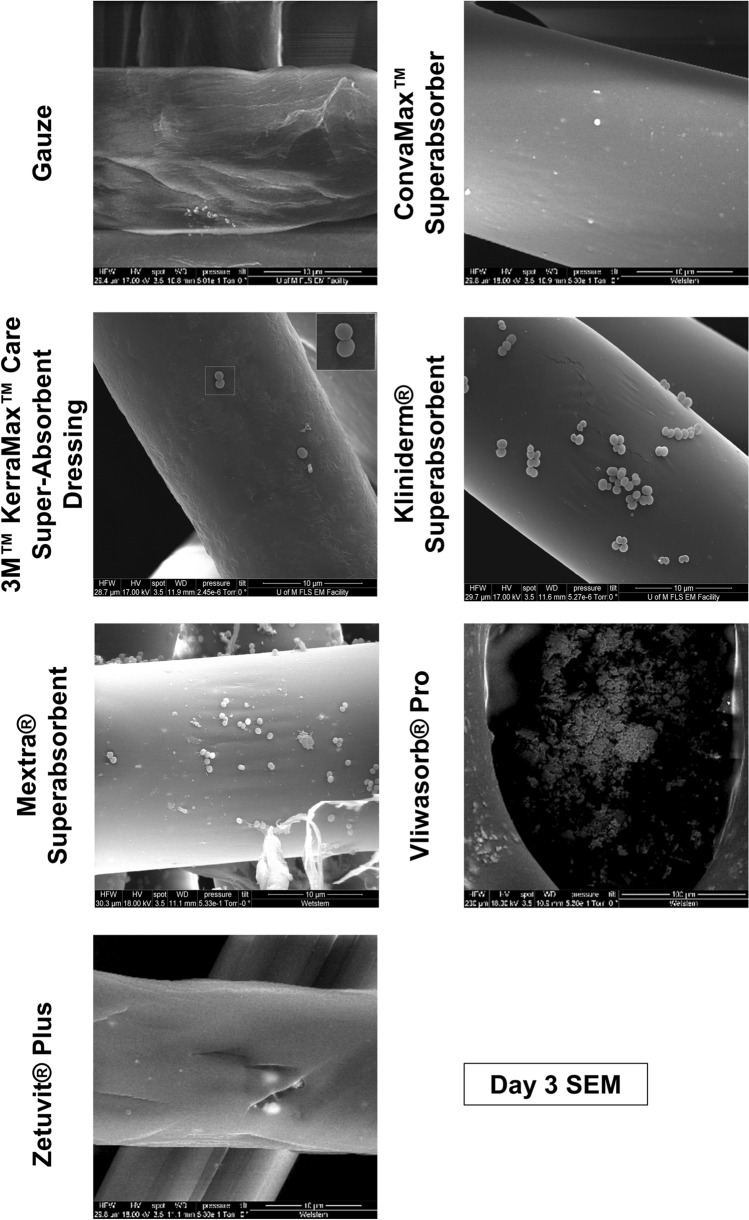
Localisation of *S. aureus* within the outer layer of superabsorbent dressings at day 3. Superabsorbent wound dressings were inoculated daily with 1 * 10^7^ CFU/ml of either *Staphylococcus aureus* over 7 days in triplicate. On 1, 3 and 7 days of inoculation, the outer membrane of each dressing was removed and fixed in glutaraldehyde, before being critical point dried, painted with conductive silver and sputter coated. Representative images of the outer membrane for dressings inoculated for 3 days with *S. aureus* are shown. Figure produced in Microsoft PowerPoint 365.

Bacteria that reside on the wound contact layer are in direct contact with the wound, increasing the risk of infection. We were therefore interested in understanding the nature of bacterial localisation at this point of contact. We imaged the wound contact layer of the superabsorbent dressings at each time point via SEM. On day 3, when inoculated with *S. aureus* (Fig. 7), the gauze control appeared surprisingly devoid of bacteria. In contrast, the outer layer of Kliniderm Superabsorbent and Mextra Superabsorbent was saturated with *S.aureus*, whereas Kerramax Care Dressing, ConvaMax Superabsorber and Zetuvit Plus had far fewer and isolated colonies. The composition of the outer layer of Vliwasorb Pro was considerably different to the other dressings, with a smooth surface and pitted holes, in which *S. aureus* appeared to localise. On day 1 and day 7 (Supplementary Fig. 1), gauze was relatively free of bacteria. Kerramax Care Dressing, Zetuvit Plus and Mextra Superabsorbent also performed well in this regard. Although ConvaMax Superabsorber was clear of bacteria at day 1, there were considerably more colonies that could be visualised at day 7. Vliwasorb Pro had possible bacterial structural formation at day 1 and isolated colonies could be seen at day 7.

**Figure 8:**
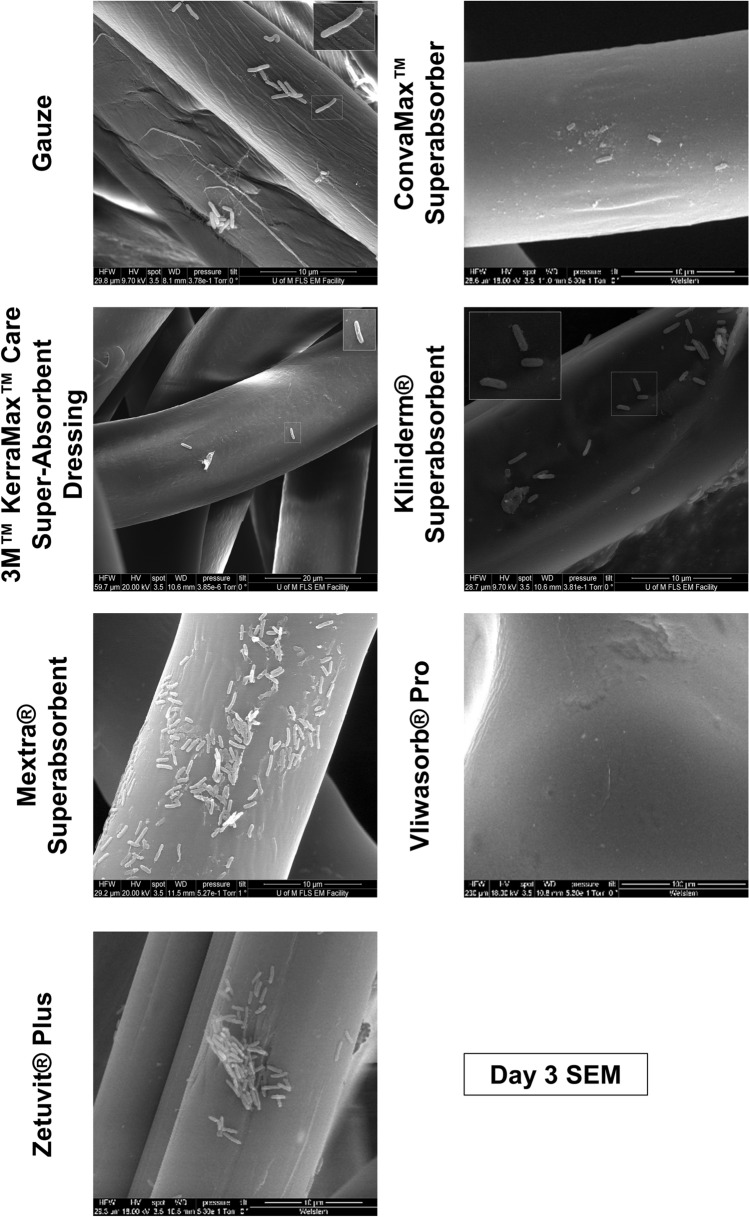
Localisation of *P. aeruginosa* within the outer layer of superabsorbent dressings at day 3. Superabsorbent wound dressings were inoculated daily with 1 * 10^7^ CFU/ml of either *Pseudomonas aeruginosa* daily, over 7 days in triplicate. On 1, 3 and 7 days of inoculation, the outer membrane of each dressing was removed and fixed in glutaraldehyde, before being critical point dried, painted with conductive silver and sputter coated. Representative images of the outer membrane for dressings inoculated for 3 days with *P. aeruginosa*, are shown. Figure produced in Microsoft PowerPoint 365.

With respect to *P. aeruginosa* at day 3 (Fig. 8), gauze, Zetuvit Plus and Mextra Superabsorbent appeared saturated with bacteria. Considerably fewer colonies could be identified on all other dressings tested. At day 1 and 7 (Supplementary Fig. 2), gauze was saturated with bacteria at day 1 and fewer bacteria were visible at day 7. Kerramax Care Dressing, ConvaMax Superabsorber, Vliwasorb Pro and Zetuvit Plus all performed well at day 1, in comparison to Kliniderm Superabsorbent where many colonies were visible and Mextra Superabsorbent, where biofilm formation could be seen. The outer layer of all dressings was saturated with *P. aeruginosa* at day 7. Thus, *P. aeruginosa* was comparatively more effective than *S. aureus* at colonising dressings*.*

### Sequestration of MMPs by superabsorbent dressings

**Figure 9:**
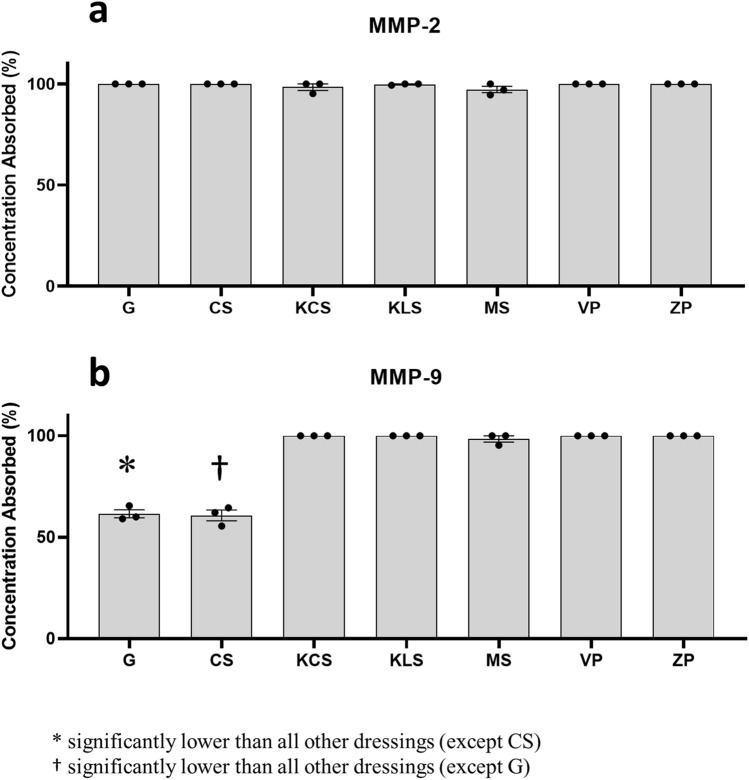
Absorption of MMPs by wound dressings. Wound dressings were inoculated with a solution of either MMP-2 or 9 at 6 ng/ml. Dressings were incubated for 4 days, the supernatant collected and the remaining concentration of each MMP was determined via enzyme-linked immunosorbent assay (ELISA). The following dressings were tested: Gauze (G), ConvaMax Superabsorber (CS), 3M Kerramax Care Super-Absorbent Dressing (KCS), Kliniderm Superabsorbent (KLS), Mextra Superabsorbent (MS), Vliwasorb Pro (VP) and Zetuvit Plus (ZP). Data for MMP-2 (**a**) and MMP-9 (**b**) is shown as percentage MMP absorbed (+ /− SEM, n = 3). Figure produced in GraphPad Prism V8.

Dysregulated production of factors such as MMPs found in wound exudate, also contributes to tissue damage and impaired healing in highly exuding wounds. To address this, we investigated the ability of these dressings to sequester two key MMPs: MMP-2 and MMP-9 (Fig. 9). MMP-2 was absorbed by all dressings (Fig. 9a). The findings for MMP-9 (Fig. 9b) were akin to the data shown for MMP-2, except for ConvaMax Superabsorber and the gauze control which had the lowest absorption (~ 60%).

## Discussion

In recent years there has been a significant increase in the number of commercially available superabsorbent dressings which are used by healthcare providers to manage moderate to highly exuding wounds. This study provides healthcare workers with a comprehensive comparison of the effectiveness of various dressings to absorb and retain wound exudate and its harmful components. We have summarised the performance of each dressing (Table 2).

**Table 2:**
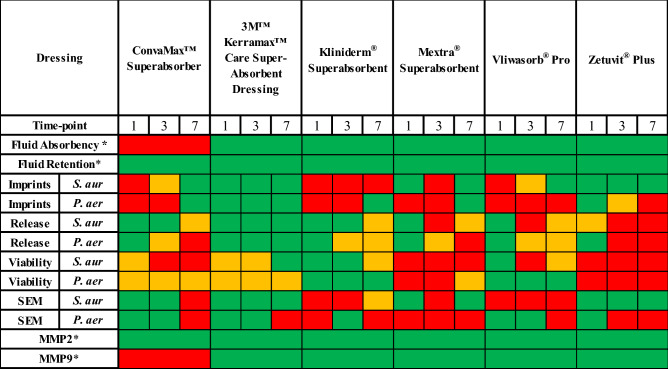
Summary table of dressing performance.

Green indicates comparatively good performance, amber indicates average performance and red indicates comparatively poor performance.

*represents a single time point.

The ability of these dressings to retain large volumes is universal as shown by the absorbency data (Fig. 1). Indeed, these dressings are designed to have fluid handling properties greater than the volume released by a highly exuding wound. As these dressings vary in size we compared the absorbency in g/100 cm^2^ based on two different measurements: the overall size of the dressing and the size of the superabsorbent inner core. The inner core is often much smaller than the overall dressing size to allow the core to swell as it absorbs fluid and thus reduces the risk of the dressing splitting open. We found that all dressings absorbed a significant amount of fluid. Importantly, the absorbency of a dressing is irrelevant if it has poor retentive properties. The effectiveness of the dressings to retain fluid varied significantly, with Vliwasorb Pro and Kerramax Care Dressing retaining the most amount of fluid. In contrast, some dressings released up to a third of the fluid they absorbed when placed under 40 mmHg. This increases the risk of maceration. Although there are some discrepancies when compared against a previous study that has investigated similar dressings^9^, such as a difference in the total absorbence for Kliniderm Superabsorbent, it is possible that there may be differences in methodology. For instance, we have used a salt solution, whereas the aforementioned study used water^9^. In either case, wound exudate consists of an array of large molecules^10^ and so it is possible that the fluid handling and retention properties of each dressing may be different in situ. Thus, the composition of the fluid used in these tests is an important consideration for future work. The size of the dressing is also important and in this study, dressing area was calculated based on the inner core of the dressing and the overall size of the dressing to clarify the different methods of determining the fluid handling properties of the dressings. Our study also investigates the ability of these highly absorbent dressings to sequester and retain bacteria. We found clear differences between dressings in their ability to transfer bacteria onto agar, indicating that the bacteria were either not absorbed into the inner core of the dressing and remained on the wound contact surface or that the bacteria was not locked away within the inner core (Figs. 2, 3).

We also found significant differences in bacteria released from the superabsorbent core when physically disrupted (Fig. 4), as well as differences in the viability of these bacteria within the inner core (Figs. 5, 6). The difference in bacterial release between dressings is intriguing, as it would highlight that the superabsorbent technology in some of the dressings is more effective at internalising the bacteria. Indeed, the dressing material and the superabsorbent polymers themselves can differ, with the efficacy of bacterial cellulose as an absorbent polymer being recently investigated^27^. Regardless, the choice of dressing must be capable of absorbing large volumes of exudate which all superabsorbent dressings tested in this study could do, with varying effectiveness, in order to sequester bacteria away from the wound.

The proportions of viable and dead bacteria within each dressing differed (Figs. 5, 6). None of these dressings have direct antimicrobial properties and so the most probable cause of bacterial death is via internalisation and limiting access to nutrients and growth factors or through dessication. Surprisingly, gauze released higher proportions of dead bacteria than expected. It is possible that gauze’s limited capacity for fluid retention may lead to faster dessication over time. However, these data contrast with results for bacterial transfer (Figs. 2, 3) and release (Fig. 4a,b), where gauze and other dressings release large concentrations of viable bacteria that is not reflected in the flow cytometry data. This could be a consequence of the fact that the flow cytometry data is based on a snapshot of 10,000 bacteria, that may not fully capture the proportional breakdown of the overall population. Specifically, dressings that released more bacteria as identified by plate counts (Fig. 4a,b) are perhaps more likely to shed bacteria upon lysis. Furthermore, the absolute quantity of bacteria in each dressing can vary, and so some dressings may release proportionally more dead bacteria that are overrepresented in the flow cytometry. Therefore, the release of high proportions of dead bacteria by some dressings may not always be a positive trait in this context.

The outer layer of the dressing is potentially as important as the absorbent inner core, as bacteria may reside on the outer, wound contact layer. Curiously, gauze, on microscopic inspection by SEM, had fewer bound bacteria than the superabsorbent dressings in most instances. However, the most likely explanation for this is that the bacteria do not bind as strongly to the gauze fibres and are therefore washed away during fixation and processing. This is supported by the fact that gauze releases the largest concentration of bacteria at each time point, which would suggest that even minimal handling would be sufficient to dislodge bacteria from the gauze. It should be noted that modern woundcare often uses alternative dressings, such as hydrogel^28^ and foam based dressings, depending on the level of exudate^29^. However, hydrogel dressings are typically used for dry wounds and require an additional covering due to their non-occlusive nature^30^. There are also several variations of foam-based dressings, with some combining superabsorbent technology^29^. It would therefore be of interest to compare the superabsorbent dressings, with these modern technologies as a control dressing.

With respect to the superabsorbent dressings, there was variation in bacterial localisation on the wound contact layer. This could be due to differences in the dressing technology, where some dressings are more effective at internalising bacteria within the inner core and therefore, away from the wound contact layer. More bacteria were seen at later time points and although dressings should not be left until saturated, there may be situations where dressings may be left for extended periods without being changed, for instance if a patient has limited access to healthcare services or if the wound condition becomes more severe. Our data would suggest that in these instances, the dressings could increase the risk of infection as they absorb greater amounts of exudate. Our data also highlights that *P. aeruginosa* was more abundant in the outer layer of dressings compared to *S. aureus* and was also more readily transferred to agar. This could be due to the array of motility mechanisms in *P. aeruginosa*^31^, in comparison to *S. aureus*, where mechanisms have only recently been defined^32^. Thus, *P. aeruginosa* may be better adapted for colonising a wound dressing over time. These bacteria also have documented interactions with each other, particularly in the context of biofilms^33,34^ and exploring this idea further, using co-cultures of bacteria, would be relevant to wound colonisation.

The absorptive properties of wound dressings are important not only for internalising bacteria, but also for the sequestering of host-derived enzymes, that may impair wound healing. Previous studies investigating MMP-2 and MMP-9 absorption by a superabsorbent dressing showed significant absorption compared to the control^20,35^. In the present study, gauze was used as a control as it does not have superabsorbent properties but showed clear efficacy in sequestering MMP-2. The previous studies by Wiegand et al.^20,35^ stipulate that the superabsorbent polymers contained in the dressing are sufficient to bind and sequester enzymes, however, it could be that many fabrics capable of absorbing fluid could have this ability. Indeed, a glass coverslip was used as a highly effective negative control in those studies, but would not rule out other, non-absorbent dressings, as absorbers of MMPs. Wiegand et al*.*^20,35^ also showed that collagenase activity was mostly inhibited by the superabsorbent dressing, although residual activity could be detected from bound collagenase. Gauze could also have this inhibitory effect, but this may not be relevant in context, as best clinical practice for managing a highly exuding wound would involve the use of superabsorbent dressings. Regardless, the fact that non-superabsorbent fabrics could inhibit enzymatic activity may be useful in areas where healthcare is limited. Although we did not assess enzymatic activity in this study, it could be a key area of investigation for future work.

The parameters investigated in the current study are pertinent to wound healing, however, it would be important to consider the dressing performance in vivo. For instance, what impact does the dressing have on the wound and the skin itself? Superabsorbent dressings have been used safely in the clinic for many years, and studies have previously shown that antimicrobial dressings in vivo can improve healing outcome^36^. However, investigating superabsorbent dressings in vivo*,* in the context of bacterial infection would be particularly important, especially when examining prevention of biofilm formation. Additionally, in an in vivo wound, exudate delivery would happen slowly over time and therefore the performance of the dressings may change in this environment. It has been reported that superabsorbent dressings can reduce tissue maceration through exudate management^37^ and there have been isolated clinical studies testing individual dressings such as Vliwasorb Pro^38^. However, a large scale comparative study in vivo would be necessary to determine the efficacy of each dressing in context.

Overall, superabsorbent dressings were considerably more effective than non-superabsorbent dressings such as gauze, with respect to bacterial burden and saturation. However, there is great variability in the efficacy of superabsorbent dressings. Some dressings are particularly effective at internalising bacteria and preventing transfer, as well as maintaining consistently low bacterial numbers despite repeated inoculations. This study gives a comprehensive comparison of the fluid, bacterial and enzyme-handling and retention properties of superabsorbent dressings, which will aid healthcare workers when selecting an appropriate superabsorbent dressing for the condition of the wound.

## Supplementary Information

Supplementary Information 1.

Supplementary Information 2.
