# Supplementary material for: Investigating the microbial and metalloprotease sequestration properties of superabsorbent wound dressings
Source: Sci Rep. 2022 Mar 19;12:4747. doi: 10.1038/s41598-022-08361-3 (PMC8934342; doi:10.1038/s41598-022-08361-3)
Supplement: Supplementary file 1 — Supplementary Information 1. [file 41598_2022_8361_MOESM1_ESM.pdf]

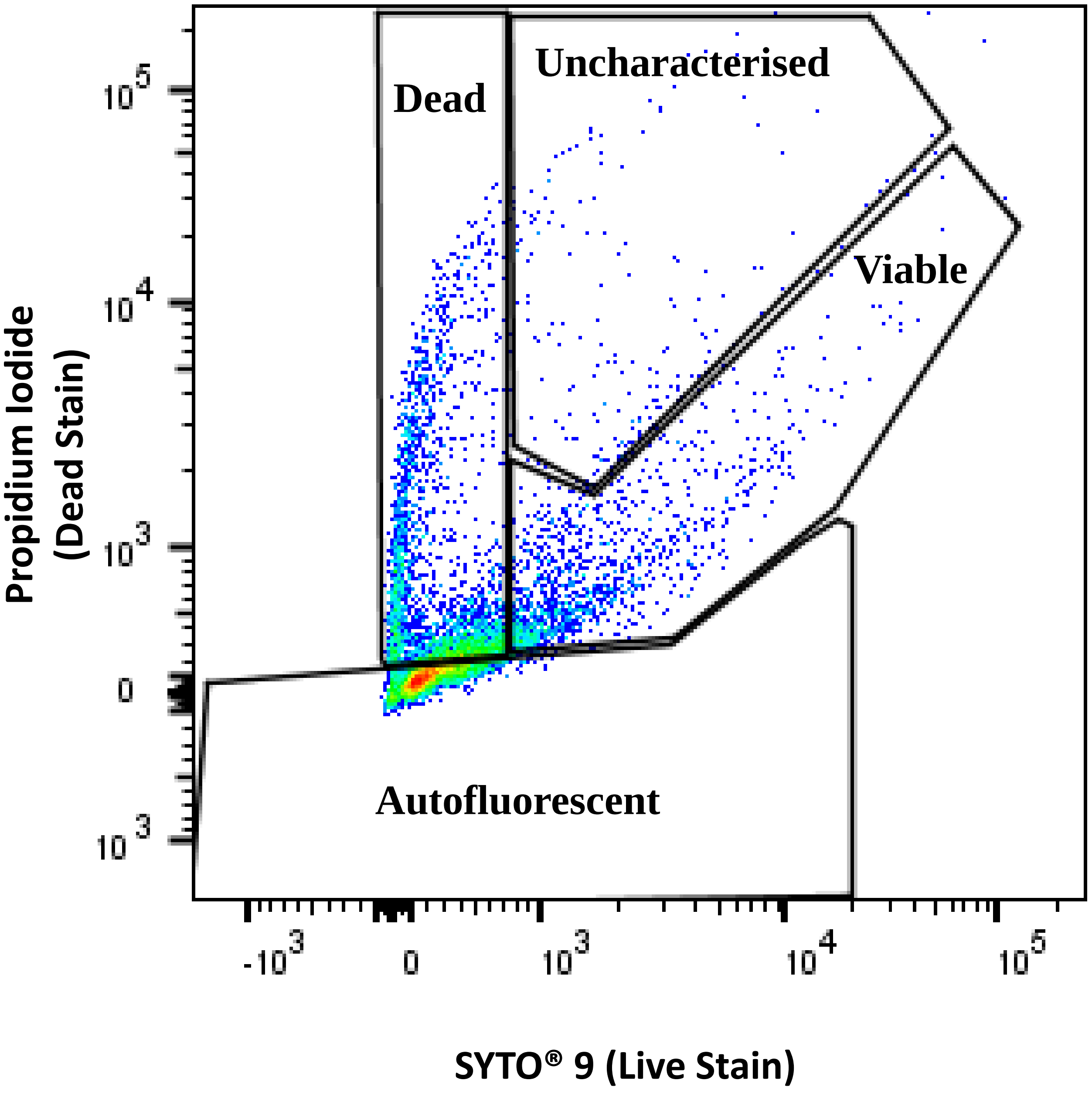

Supplementary Figure 1: Gating strategy for bacterial viability experiments.

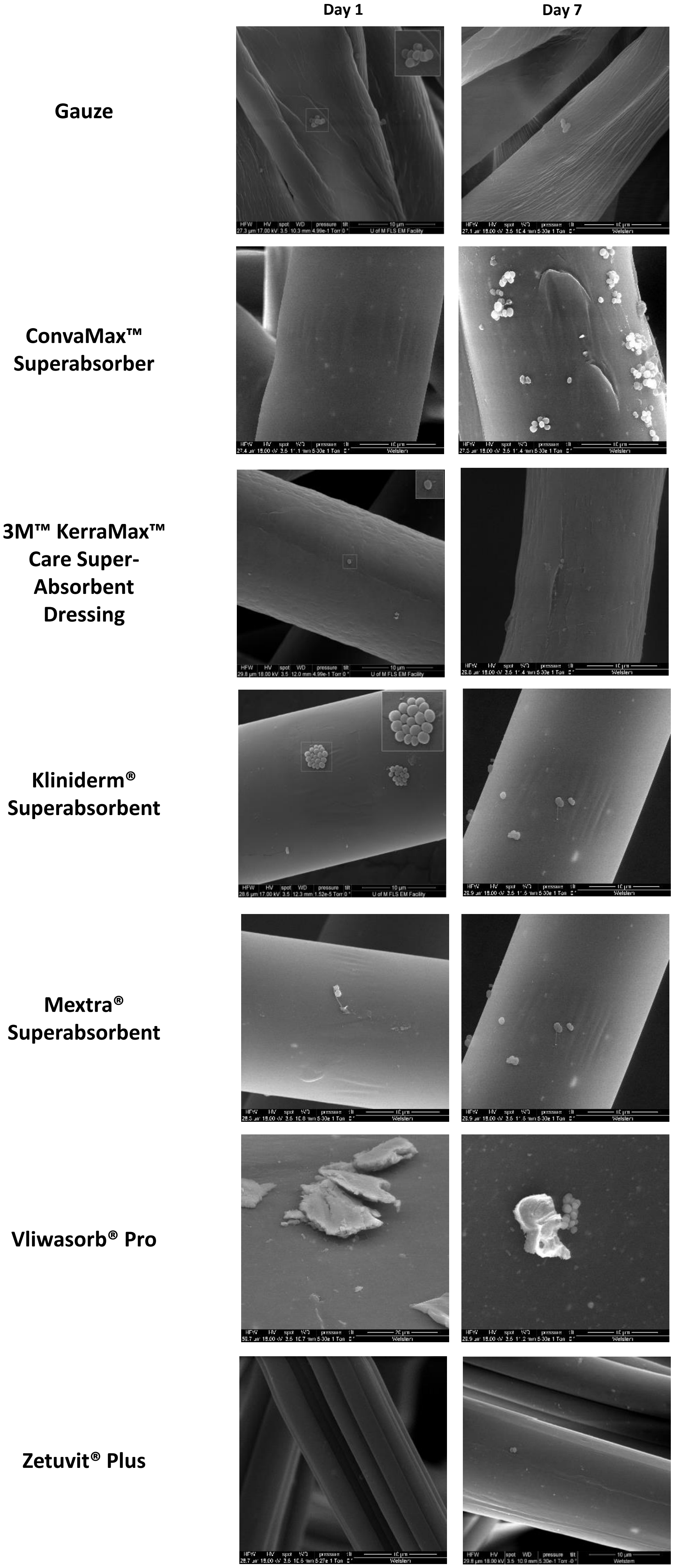

Supplementary Figure 2: Localisation of *S. aureus* within outer layer of superabsorbent dressings at day 1 and 7. Commercially available superabsorbent wound dressings were inoculated with 1 * 10^7 CFU/ml of either *Staphylococcus aureus* daily, over 7 days in triplicate. At 1, 3 and 7 days of inoculation, the outer membrane of each dressing was removed and fixed in glutaraldehyde, before being critical point dried, painted with conductive silver and sputter coated. Representative images of the outer membrane for dressings inoculated with *S. aureus* for 1 and 7 days, are shown.

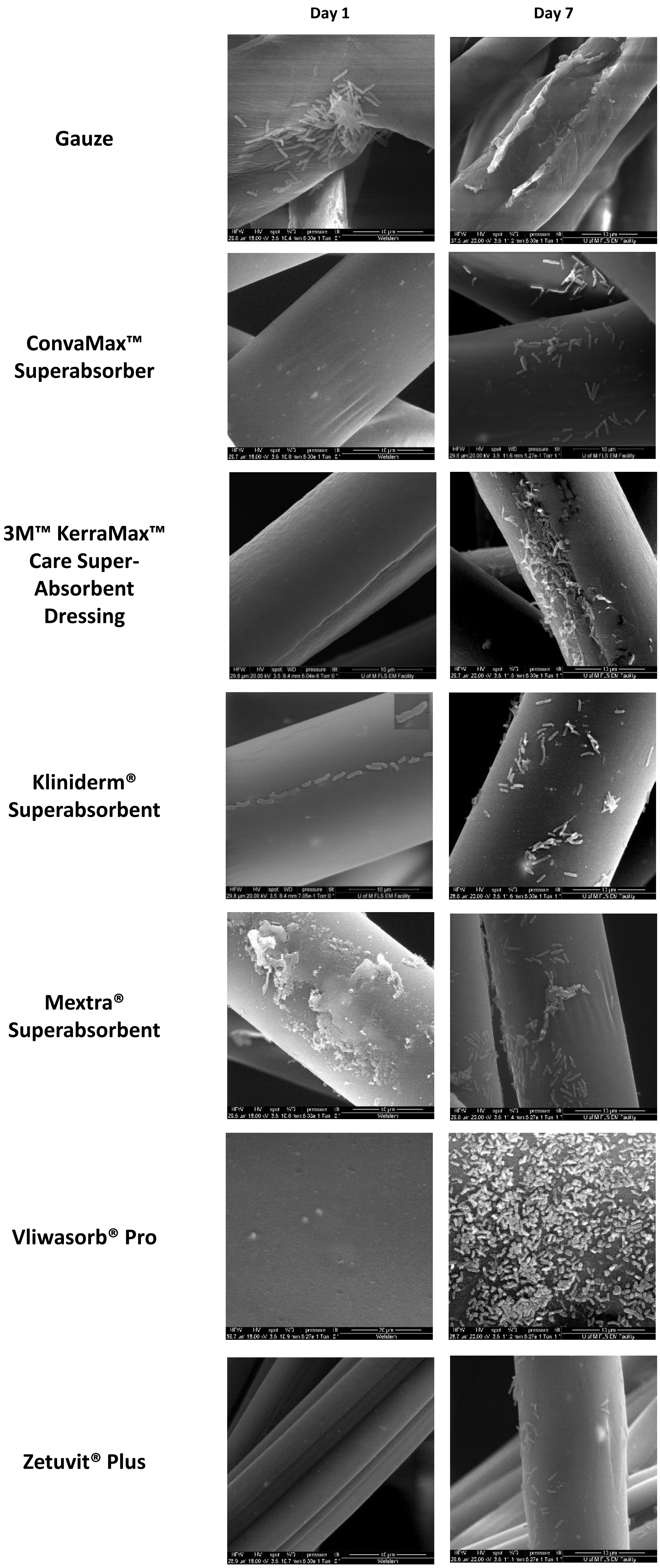

Supplementary Figure 3: Localisation of *P. aeruginosa* within outer layer of superabsorbent dressings at day 1 and 7. Commercially available wound dressings were inoculated with 1 * 10^7 CFU/ml of either *Pseudomonas aeruginosa* daily, over 7 days in triplicate. At 1, 3 and 7 days of inoculation, the outer membrane of each dressing was removed and fixed in glutaraldehyde, before being critical point dried, painted with conductive silver and sputter coated. Representative images of the outer membrane for dressings inoculated with *P. aeruginosa* for 1 and 7 days, are shown.
