# Supplementary material for: Investigating the microbial and metalloprotease sequestration properties of superabsorbent wound dressings
Source: Sci Rep. 2022 Mar 19;12:4747. doi: 10.1038/s41598-022-08361-3 (PMC8934342; doi:10.1038/s41598-022-08361-3)
Supplement: Supplementary file 2 — Supplementary Information 2. [file 41598_2022_8361_MOESM2_ESM.pdf]

Supplementary Table 1: Significant differences for fluid absorption data (Figure 1a).

Comparison [a]	Type	Significance [b]	Higher/Lower [c]	P Value
CS vs. KCS	Core only	****	Lower	<0.0001
CS vs. KLS	Core only	****	Lower	<0.0001
CS vs. MS	Core only	****	Lower	<0.0001
CS vs. VP	Core only	ns	N/A	0.9341
CS vs. ZP	Core only	****	Lower	<0.0001
KCS vs. KLS	Core only	****	Lower	<0.0001
KCS vs. MS	Core only	***	Lower	0.0001
KCS vs. VP	Core only	****	Higher	<0.0001
KCS vs. ZP	Core only	****	Higher	<0.0001
KLS vs. MS	Core only	****	Higher	<0.0001
KLS vs. VP	Core only	****	Higher	<0.0001
KLS vs. ZP	Core only	****	Higher	<0.0001
MS vs. VP	Core only	****	Higher	<0.0001
MS vs. ZP	Core only	****	Higher	<0.0001
VP vs. ZP	Core only	****	Lower	<0.0001
CS vs. KCS	Full dressing	****	Lower	<0.0001
CS vs. KLS	Full dressing	****	Lower	<0.0001
CS vs. MS	Full dressing	****	Lower	<0.0001
CS vs. VP	Full dressing	****	Lower	<0.0001
CS vs. ZP	Full dressing	****	Lower	<0.0001
KCS vs. KLS	Full dressing	ns	N/A	0.5732
KCS vs. MS	Full dressing	ns	N/A	0.6717
KCS vs. VP	Full dressing	***	Higher	0.0009
KCS vs. ZP	Full dressing	ns	N/A	0.0655
KLS vs. MS	Full dressing	*	Lower	0.0304
KLS vs. VP	Full dressing	ns	N/A	0.0998
KLS vs. ZP	Full dressing	ns	N/A	0.8397
MS vs. VP	Full dressing	****	Higher	<0.0001
MS vs. ZP	Full dressing	***	Higher	0.0008
VP vs. ZP	Full dressing	ns	N/A	0.6873

a ConvaMax™ Superabsorber (CS), 3M™ Kerramax™ Care Super-Absorbent Dressing (KCS), Kliniderm® Superabsorbent (KLS), Mextra® Superabsorbent (MS), Vliwasorb® Pro (VP), and Zetuvit® Plus (ZP).

b not significant (ns).

c significantly higher or lower absorbance compared to the other dressing.

Supplementary Table 2: Significant differences for fluid retention/release data at 30 mmHg (Figure 1b).

Comparison [a]	Type	Significance [b]	Higher/Lower [c]	P Value
CS vs. KCS	Retention/Release	****	Lower	<0.0001
CS vs. KLS	Retention/Release	****	Lower	<0.0001
CS vs. MS	Retention/Release	ns	N/A	0.1457
CS vs. VP	Retention/Release	****	Lower	<0.0001
CS vs. ZP	Retention/Release	**	Higher	0.0014
KCS vs. KLS	Retention/Release	****	Higher	<0.0001
KCS vs. MS	Retention/Release	****	Higher	<0.0001
KCS vs. VP	Retention/Release	***	Lower	0.0009
KCS vs. ZP	Retention/Release	****	Higher	<0.0001
KLS vs. MS	Retention/Release	****	Higher	<0.0001
KLS vs. VP	Retention/Release	****	Lower	<0.0001
KLS vs. ZP	Retention/Release	****	Higher	<0.0001
MS vs. VP	Retention/Release	****	Lower	<0.0001
MS vs. ZP	Retention/Release	****	Higher	<0.0001
VP vs. ZP	Retention/Release	****	Higher	<0.0001

a ConvaMax™ Superabsorber (CS), 3M™ Kerramax™ Care Super-Absorbent Dressing (KCS), Kliniderm® Superabsorbent (KLS), Mextra® Superabsorbent (MS), Vliwasorb® Pro (VP), and Zetuvit® Plus (ZP).

b not significant (ns).

c significantly higher or lower retention compared to the other dressing.

Supplementary Table 3: Significant differences for fluid retention/release data at 40 mmHg (Figure 1c).

Comparison [a]	Type	Significance [b]	Higher/Lower [c]	P Value
CS vs. KCS	Retention/Release	****	Lower	<0.0001
CS vs. KLS	Retention/Release	****	Lower	<0.0001
CS vs. MS	Retention/Release	ns	Lower	0.3097
CS vs. VP	Retention/Release	****	Lower	<0.0001
CS vs. ZP	Retention/Release	****	Higher	<0.0001
KCS vs. KLS	Retention/Release	****	Higher	<0.0001
KCS vs. MS	Retention/Release	****	Higher	<0.0001
KCS vs. VP	Retention/Release	****	Lower	<0.0001
KCS vs. ZP	Retention/Release	****	Higher	<0.0001
KLS vs. MS	Retention/Release	***	Higher	0.0002
KLS vs. VP	Retention/Release	****	Lower	<0.0001
KLS vs. ZP	Retention/Release	****	Higher	<0.0001
MS vs. VP	Retention/Release	****	Lower	<0.0001
MS vs. ZP	Retention/Release	****	Higher	<0.0001
VP vs. ZP	Retention/Release	****	Higher	<0.0001

a ConvaMax™ Superabsorber (CS), 3M™ Kerramax™ Care Super-Absorbent Dressing (KCS), Kliniderm® Superabsorbent (KLS), Mextra® Superabsorbent (MS), Vliwasorb® Pro (VP), and Zetuvit® Plus (ZP).

b not significant (ns).

c significantly higher or lower retention compared to the other dressing.

Supplementary Table 4: Significant differences for *S. aureus* release at day 3 (Figure 4a).

Comparison [a]	Type	Significance [b]	Higher/Lower [c]	P Value
G vs. CS	*S. aureus* Release	*	Higher	0.0131
G vs. KCS	*S. aureus* Release	*	Higher	0.0126
G vs. KL	*S. aureus* Release	*	Higher	0.0125
G vs. MS	*S. aureus* Release	*	Higher	0.0390
G vs. VP	*S. aureus* Release	ns	N/A	>0.9999
G vs. ZP	*S. aureus* Release	ns	N/A	0.0626
CS vs. KCS	*S. aureus* Release	ns	N/A	>0.9999
CS vs. KLS	*S. aureus* Release	ns	N/A	>0.9999
CS vs. MS	*S. aureus* Release	ns	N/A	0.9999
CS vs. VP	*S. aureus* Release	**	Lower	0.0083
CS vs. ZP	*S. aureus* Release	ns	N/A	0.9986
KCS vs. KLS	*S. aureus* Release	ns	N/A	>0.9999
KCS vs. MS	*S. aureus* Release	ns	N/A	0.9999
KCS vs. VP	*S. aureus* Release	**	Lower	0.0080
KCS vs. ZP	*S. aureus* Release	ns	N/A	0.9984
KLS vs. MS	*S. aureus* Release	ns	N/A	0.9999
KLS vs. VP	*S. aureus* Release	**	Lower	0.0079
KLS vs. ZP	*S. aureus* Release	ns	N/A	0.9983
MS vs. VP	*S. aureus* Release	*	Lower	0.0256
MS vs. ZP	*S. aureus* Release	ns	N/A	>0.9999
VP vs. ZP	*S. aureus* Release	*	Lower	0.0419

a ConvaMax™ Superabsorber (CS), 3M™ Kerramax™ Care Super-Absorbent Dressing (KCS), Kliniderm® Superabsorbent (KLS), Mextra® Superabsorbent (MS), Vliwasorb® Pro (VP), and Zetuvit® Plus (ZP).

b not significant (ns).

c significantly higher or lower release compared to the other dressing.

Supplementary Table 5: Significant differences for *P. aeruginosa* release at day 3 and 7 (Figure 4b).

Comparison [a]	Type	Significance [b]	Higher/Lower [c]	P Value
G vs. CS	*P. aeruginosa* Release	ns	N/A	0.5145
G vs. KCS	*P. aeruginosa* Release D3	*	Higher	<0.0483
G vs. KCS	*P. aeruginosa* Release	****	Higher	<0.0001
G vs. KLS	*P. aeruginosa* Release	****	Higher	<0.0001
G vs. MS	*P. aeruginosa* Release	**	Higher	0.0019
G vs. VP	*P. aeruginosa* Release	****	Higher	<0.0001
G vs. ZP	*P. aeruginosa* Release	ns	N/A	0.1460
CS vs. KCS	*P. aeruginosa* Release	****	Higher	<0.0001
CS vs. KLS	*P. aeruginosa* Release	****	Higher	<0.0001
CS vs. MS	*P. aeruginosa* Release	ns	N/A	0.2700
CS vs. VP	*P. aeruginosa* Release	****	Higher	<0.0001
CS vs. ZP	*P. aeruginosa* Release	ns	N/A	0.9948
KCS vs. KLS	*P. aeruginosa* Release	ns	N/A	0.9999
KCS vs. MS	*P. aeruginosa* Release	*	Lower	0.0126
KCS vs. VP	*P. aeruginosa* Release	ns	N/A	>0.9999
KCS vs. ZP	*P. aeruginosa* Release	****	Lower	<0.0001
KLS vs. MS	*P. aeruginosa* Release	*	Lower	0.0382
KLS vs. VP	*P. aeruginosa* Release	ns	N/A	>0.9999
KLS vs. ZP	*P. aeruginosa* Release	***	Lower	0.0003
MS vs. VP	*P. aeruginosa* Release	*	Higher	0.0331
MS vs. ZP	*P. aeruginosa* Release	ns	N/A	0.7196
VP vs. ZP	*P. aeruginosa* Release	***	Lower	0.0003

a ConvaMax™ Superabsorber (CS), 3M™ Kerramax™ Care Super-Absorbent Dressing (KCS), Kliniderm® Superabsorbent (KLS), Mextra® Superabsorbent (MS), Vliwasorb® Pro (VP), and Zetuvit® Plus (ZP).

b not significant (ns).

c significantly higher or lower release compared to the other dressing.

Supplementary Table 6: Significant differences for *S. aureus* viability at day 1 (Figure 5a).

Comparison [a]	Type	Significance [b]	Higher/Lower [c]	P Value
B vs. G	Proportion Dead	****	Lower	<0.0001
B vs. CM	Proportion Dead	****	Lower	<0.0001
B vs. KCS	Proportion Dead	****	Lower	<0.0001
B vs. KLS	Proportion Dead	****	Lower	<0.0001
B vs. M	Proportion Dead	****	Lower	<0.0001
B vs. VP	Proportion Dead	****	Lower	<0.0001
B vs. ZP	Proportion Dead	ns	Lower	0.5754
G vs. CM	Proportion Dead	ns	Lower	0.1756
G vs. KCS	Proportion Dead	ns	Lower	0.1772
G vs. KLS	Proportion Dead	****	Lower	<0.0001
G vs. M	Proportion Dead	ns	Lower	0.7839
G vs. VP	Proportion Dead	****	Lower	<0.0001
G vs. ZP	Proportion Dead	****	Higher	<0.0001
CM vs. KCS	Proportion Dead	ns	NA	>0.9999
CM vs. KLS	Proportion Dead	***	Lower	0.0001
CM vs. M	Proportion Dead	**	Higher	0.0046
CM vs. VP	Proportion Dead	*	Lower	0.0388
CM vs. ZP	Proportion Dead	****	Higher	<0.0001
KCS vs. KLS	Proportion Dead	***	Lower	0.0001
KCS vs. M	Proportion Dead	**	Higher	0.0046
KCS vs. VP	Proportion Dead	*	Lower	0.0383
KCS vs. ZP	Proportion Dead	****	Higher	<0.0001
KLS vs. M	Proportion Dead	****	Higher	<0.0001
KLS vs. VP	Proportion Dead	ns	Higher	0.4011
KLS vs. ZP	Proportion Dead	****	Higher	<0.0001
M vs. VP	Proportion Dead	****	Lower	<0.0001
M vs. ZP	Proportion Dead	**	Higher	0.0012
VP vs. ZP	Proportion Dead	****	Higher	<0.0001
B vs. G	Proportion Viable	****	Higher	<0.0001
B vs. CM	Proportion Viable	****	Higher	<0.0001
B vs. KCS	Proportion Viable	****	Higher	<0.0001
B vs. KLS	Proportion Viable	****	Higher	<0.0001
B vs. M	Proportion Viable	****	Higher	<0.0001
B vs. VP	Proportion Viable	****	Higher	<0.0001
B vs. ZP	Proportion Viable	ns	Higher	0.5754
G vs. CM	Proportion Viable	ns	Higher	0.1756
G vs. KCS	Proportion Viable	ns	Higher	0.1772
G vs. KLS	Proportion Viable	****	Higher	<0.0001
G vs. M	Proportion Viable	ns	Lower	0.7839
G vs. VP	Proportion Viable	****	Higher	<0.0001
G vs. ZP	Proportion Viable	****	Lower	<0.0001
CM vs. KCS	Proportion Viable	ns	NA	>0.9999
CM vs. KLS	Proportion Viable	***	Higher	0.0001
CM vs. M	Proportion Viable	**	Lower	0.0046
CM vs. VP	Proportion Viable	*	Higher	0.0388
CM vs. ZP	Proportion Viable	****	Lower	<0.0001
KCS vs. KLS	Proportion Viable	***	Higher	0.0001
KCS vs. M	Proportion Viable	**	Lower	0.0046
KCS vs. VP	Proportion Viable	*	Higher	0.0383
KCS vs. ZP	Proportion Viable	****	Lower	<0.0001
KLS vs. M	Proportion Viable	****	Lower	<0.0001
KLS vs. VP	Proportion Viable	ns	Lower	0.4011
KLS vs. ZP	Proportion Viable	****	Lower	<0.0001
M vs. VP	Proportion Viable	****	Higher	<0.0001
M vs. ZP	Proportion Viable	**	Lower	0.0012
VP vs. ZP	Proportion Viable	****	Lower	<0.0001

a Broth (B), ConvaMax™ Superabsorber (CS), 3M™ Kerramax™ Care Super-Absorbent Dressing (KCS), Kliniderm® Superabsorbent (KLS), Mextra® Superabsorbent (MS), Vliwasorb® Pro (VP), and Zetuvit® Plus (ZP).

b not significant (ns).

c significantly (or a trend towards) higher or lower proportion compared to the other dressing.

Supplementary Table 7: Significant differences for *S. aureus* viability at day 3 (Figure 5b).

Comparison [a]	Type	Significance [b]	Higher/Lower [c]	P Value
B vs. G	Proportion Dead	****	Lower	<0.0001
B vs. CM	Proportion Dead	***	Lower	0.0002
B vs. KCS	Proportion Dead	****	Lower	<0.0001
B vs. KLS	Proportion Dead	****	Lower	<0.0001
B vs. M	Proportion Dead	***	Lower	0.0005
B vs. VP	Proportion Dead	****	Lower	<0.0001
B vs. ZP	Proportion Dead	**	Lower	0.0012
G vs. CM	Proportion Dead	***	Higher	0.0003
G vs. KCS	Proportion Dead	ns	Higher	0.1471
G vs. KLS	Proportion Dead	ns	Lower	0.8499
G vs. M	Proportion Dead	***	Higher	0.0001
G vs. VP	Proportion Dead	**	Higher	0.0033
G vs. ZP	Proportion Dead	****	Higher	<0.0001
CM vs. KCS	Proportion Dead	ns	Lower	0.2776
CM vs. KLS	Proportion Dead	****	Lower	<0.0001
CM vs. M	Proportion Dead	ns	Higher	>0.9999
CM vs. VP	Proportion Dead	ns	Lower	0.99
CM vs. ZP	Proportion Dead	ns	Lower	0.9974
KCS vs. KLS	Proportion Dead	**	Lower	0.0051
KCS vs. M	Proportion Dead	ns	Higher	0.1495
KCS vs. VP	Proportion Dead	ns	Higher	0.7657
KCS vs. ZP	Proportion Dead	ns	Higher	0.0754
KLS vs. M	Proportion Dead	****	Higher	<0.0001
KLS vs. VP	Proportion Dead	****	Higher	<0.0001
KLS vs. ZP	Proportion Dead	****	Higher	<0.0001
M vs. VP	Proportion Dead	ns	Lower	0.9358
M vs. ZP	Proportion Dead	ns	Higher	>0.9999
VP vs. ZP	Proportion Dead	ns	Higher	0.8063
B vs. G	Proportion Viable	****	Higher	<0.0001
B vs. CM	Proportion Viable	***	Higher	0.0002
B vs. KCS	Proportion Viable	****	Higher	<0.0001
B vs. KLS	Proportion Viable	****	Higher	<0.0001
B vs. M	Proportion Viable	***	Higher	0.0005
B vs. VP	Proportion Viable	****	Higher	<0.0001
B vs. ZP	Proportion Viable	**	Higher	0.0012
G vs. CM	Proportion Viable	***	Lower	0.0003
G vs. KCS	Proportion Viable	ns	Lower	0.1471
G vs. KLS	Proportion Viable	ns	Higher	0.8499
G vs. M	Proportion Viable	***	Lower	0.0001
G vs. VP	Proportion Viable	**	Lower	0.0033
G vs. ZP	Proportion Viable	****	Lower	<0.0001
CM vs. KCS	Proportion Viable	ns	Higher	0.2776
CM vs. KLS	Proportion Viable	****	Higher	<0.0001
CM vs. M	Proportion Viable	ns	Lower	>0.9999
CM vs. VP	Proportion Viable	ns	Higher	0.99
CM vs. ZP	Proportion Viable	ns	Lower	0.9974
KCS vs. KLS	Proportion Viable	**	Higher	0.0051
KCS vs. M	Proportion Viable	ns	Lower	0.1495
KCS vs. VP	Proportion Viable	ns	Lower	0.7657
KCS vs. ZP	Proportion Viable	ns	Lower	0.0754
KLS vs. M	Proportion Viable	****	Lower	<0.0001
KLS vs. VP	Proportion Viable	****	Lower	<0.0001
KLS vs. ZP	Proportion Viable	****	Lower	<0.0001
M vs. VP	Proportion Viable	ns	Higher	0.9358
M vs. ZP	Proportion Viable	ns	Lower	>0.9999
VP vs. ZP	Proportion Viable	ns	Lower	0.8063

a Broth (B), ConvaMax™ Superabsorber (CS), 3M™ Kerramax™ Care Super-Absorbent Dressing (KCS), Kliniderm® Superabsorbent (KLS), Mextra® Superabsorbent (MS), Vliwasorb® Pro (VP), and Zetuvit® Plus (ZP).

b not significant (ns).

c significantly (or a trend towards) higher or lower proportion compared to the other dressing.

Supplementary Table 8: Significant differences for *S. aureus* viability at day 7 (Figure 5c).

Comparison [a]	Type	Significance [b]	Higher/Lower [c]	P Value
B vs. G	Proportion Dead	****	Lower	<0.0001
B vs. CM	Proportion Dead	ns	Lower	>0.9999
B vs. KCS	Proportion Dead	****	Lower	<0.0001
B vs. KLS	Proportion Dead	****	Lower	<0.0001
B vs. M	Proportion Dead	ns	Lower	0.3853
B vs. VP	Proportion Dead	****	Lower	<0.0001
B vs. ZP	Proportion Dead	ns	Lower	0.962
G vs. CM	Proportion Dead	****	Higher	<0.0001
G vs. KCS	Proportion Dead	ns	Higher	0.1243
G vs. KLS	Proportion Dead	****	Higher	<0.0001
G vs. M	Proportion Dead	****	Higher	<0.0001
G vs. VP	Proportion Dead	****	Higher	<0.0001
G vs. ZP	Proportion Dead	****	Higher	<0.0001
CM vs. KCS	Proportion Dead	****	Lower	<0.0001
CM vs. KLS	Proportion Dead	****	Lower	<0.0001
CM vs. M	Proportion Dead	ns	Lower	0.578
CM vs. VP	Proportion Dead	****	Lower	<0.0001
CM vs. ZP	Proportion Dead	ns	Lower	0.995
KCS vs. KLS	Proportion Dead	****	Higher	<0.0001
KCS vs. M	Proportion Dead	****	Higher	<0.0001
KCS vs. VP	Proportion Dead	****	Higher	<0.0001
KCS vs. ZP	Proportion Dead	****	Higher	<0.0001
KLS vs. M	Proportion Dead	****	Higher	<0.0001
KLS vs. VP	Proportion Dead	ns	Higher	0.9675
KLS vs. ZP	Proportion Dead	****	Higher	<0.0001
M vs. VP	Proportion Dead	**	Lower	0.0015
M vs. ZP	Proportion Dead	ns	Higher	0.9438
VP vs. ZP	Proportion Dead	****	Higher	<0.0001
B vs. G	Proportion Viable	****	Higher	<0.0001
B vs. CM	Proportion Viable	ns	Higher	>0.9999
B vs. KCS	Proportion Viable	****	Higher	<0.0001
B vs. KLS	Proportion Viable	****	Higher	<0.0001
B vs. M	Proportion Viable	ns	Higher	0.3853
B vs. VP	Proportion Viable	****	Higher	<0.0001
B vs. ZP	Proportion Viable	ns	Higher	0.962
G vs. CM	Proportion Viable	****	Lower	<0.0001
G vs. KCS	Proportion Viable	ns	Lower	0.1243
G vs. KLS	Proportion Viable	****	Lower	<0.0001
G vs. M	Proportion Viable	****	Lower	<0.0001
G vs. VP	Proportion Viable	****	Lower	<0.0001
G vs. ZP	Proportion Viable	****	Lower	<0.0001
CM vs. KCS	Proportion Viable	****	Higher	<0.0001
CM vs. KLS	Proportion Viable	****	Higher	<0.0001
CM vs. M	Proportion Viable	ns	Higher	0.578
CM vs. VP	Proportion Viable	****	Higher	<0.0001
CM vs. ZP	Proportion Viable	ns	Higher	0.995
KCS vs. KLS	Proportion Viable	****	Lower	<0.0001
KCS vs. M	Proportion Viable	****	Lower	<0.0001
KCS vs. VP	Proportion Viable	****	Lower	<0.0001
KCS vs. ZP	Proportion Viable	****	Lower	<0.0001
KLS vs. M	Proportion Viable	****	Lower	<0.0001
KLS vs. VP	Proportion Viable	ns	Lower	0.9675
KLS vs. ZP	Proportion Viable	****	Lower	<0.0001
M vs. VP	Proportion Viable	**	Higher	0.0015
M vs. ZP	Proportion Viable	ns	Lower	0.9438
VP vs. ZP	Proportion Viable	****	Lower	<0.0001

a ConvaMax™ Superabsorber (CS), 3M™ Kerramax™ Care Super-Absorbent Dressing (KCS), Kliniderm® Superabsorbent (KLS), Mextra® Superabsorbent (MS), Vliwasorb® Pro (VP), and Zetuvit® Plus (ZP).

b not significant (ns).

c significantly (or a trend towards) higher or lower proportion compared to the other dressing.

Supplementary Table 9: Significant differences for *P. aeruginosa* viability at day 1 (Figure 6a).

Comparison [a]	Type	Significance [b]	Higher/Lower [c]	P Value
B vs. G	Proportion Dead	ns	Lower	0.8172
B vs. CM	Proportion Dead	****	Lower	<0.0001
B vs. KCS	Proportion Dead	****	Lower	<0.0001
B vs. KLS	Proportion Dead	****	Lower	<0.0001
B vs. M	Proportion Dead	****	Lower	<0.0001
B vs. VP	Proportion Dead	****	Lower	<0.0001
B vs. ZP	Proportion Dead	***	Lower	0.0002
G vs. CM	Proportion Dead	****	Lower	<0.0001
G vs. KCS	Proportion Dead	****	Lower	<0.0001
G vs. KLS	Proportion Dead	****	Lower	<0.0001
G vs. M	Proportion Dead	****	Lower	<0.0001
G vs. VP	Proportion Dead	****	Lower	<0.0001
G vs. ZP	Proportion Dead	*	Lower	0.0103
CM vs. KCS	Proportion Dead	ns	Lower	0.4154
CM vs. KLS	Proportion Dead	****	Lower	<0.0001
CM vs. M	Proportion Dead	****	Higher	<0.0001
CM vs. VP	Proportion Dead	***	Lower	0.0004
CM vs. ZP	Proportion Dead	****	Higher	<0.0001
KCS vs. KLS	Proportion Dead	***	Lower	0.0002
KCS vs. M	Proportion Dead	****	Higher	<0.0001
KCS vs. VP	Proportion Dead	ns	Lower	0.1077
KCS vs. ZP	Proportion Dead	****	Higher	<0.0001
KLS vs. M	Proportion Dead	****	Higher	<0.0001
KLS vs. VP	Proportion Dead	ns	Higher	0.2804
KLS vs. ZP	Proportion Dead	****	Higher	<0.0001
M vs. VP	Proportion Dead	****	Lower	<0.0001
M vs. ZP	Proportion Dead	ns	Higher	0.2848
VP vs. ZP	Proportion Dead	****	Higher	<0.0001
B vs. G	Proportion Viable	ns	Higher	0.8172
B vs. CM	Proportion Viable	****	Higher	<0.0001
B vs. KCS	Proportion Viable	****	Higher	<0.0001
B vs. KLS	Proportion Viable	****	Higher	<0.0001
B vs. M	Proportion Viable	****	Higher	<0.0001
B vs. VP	Proportion Viable	****	Higher	<0.0001
B vs. ZP	Proportion Viable	***	Higher	0.0002
G vs. CM	Proportion Viable	****	Higher	<0.0001
G vs. KCS	Proportion Viable	****	Higher	<0.0001
G vs. KLS	Proportion Viable	****	Higher	<0.0001
G vs. M	Proportion Viable	****	Higher	<0.0001
G vs. VP	Proportion Viable	****	Higher	<0.0001
G vs. ZP	Proportion Viable	*	Higher	0.0103
CM vs. KCS	Proportion Viable	ns	Higher	0.4154
CM vs. KLS	Proportion Viable	****	Lower	<0.0001
CM vs. M	Proportion Viable	****	Lower	<0.0001
CM vs. VP	Proportion Viable	***	Higher	0.0004
CM vs. ZP	Proportion Viable	****	Lower	<0.0001
KCS vs. KLS	Proportion Viable	***	Higher	0.0002
KCS vs. M	Proportion Viable	****	Lower	<0.0001
KCS vs. VP	Proportion Viable	ns	Higher	0.1077
KCS vs. ZP	Proportion Viable	****	Lower	<0.0001
KLS vs. M	Proportion Viable	****	Lower	<0.0001
KLS vs. VP	Proportion Viable	ns	Lower	0.2804
KLS vs. ZP	Proportion Viable	****	Lower	<0.0001
M vs. VP	Proportion Viable	****	Higher	<0.0001
M vs. ZP	Proportion Viable	ns	Lower	0.2848
VP vs. ZP	Proportion Viable	****	Lower	<0.0001

a ConvaMax™ Superabsorber (CS), 3M™ Kerramax™ Care Super-Absorbent Dressing (KCS), Kliniderm® Superabsorbent (KLS), Mextra® Superabsorbent (MS), Vliwasorb® Pro (VP), and Zetuvit® Plus (ZP).

b not significant (ns).

c significantly (or a trend towards) higher or lower proportion compared to the other dressing.

Supplementary Table 10: Significant differences for *P. aeruginosa* viability at day 3 (Figure 6b).

Comparison [a]	Type	Significance [b]	Higher/Lower [c]	P Value
B vs. G	Proportion Dead	ns	Lower	0.8162
B vs. CM	Proportion Dead	****	Lower	<0.0001
B vs. KCS	Proportion Dead	****	Lower	<0.0001
B vs. KLS	Proportion Dead	****	Lower	<0.0001
B vs. M	Proportion Dead	**	Lower	0.0072
B vs. VP	Proportion Dead	****	Lower	<0.0001
B vs. ZP	Proportion Dead	*	Lower	0.0143
G vs. CM	Proportion Dead	****	Lower	<0.0001
G vs. KCS	Proportion Dead	****	Lower	<0.0001
G vs. KLS	Proportion Dead	****	Lower	<0.0001
G vs. M	Proportion Dead	ns	Lower	0.215
G vs. VP	Proportion Dead	****	Lower	<0.0001
G vs. ZP	Proportion Dead	ns	Lower	0.335
CM vs. KCS	Proportion Dead	ns	Lower	0.2319
CM vs. KLS	Proportion Dead	***	Lower	0.0007
CM vs. M	Proportion Dead	****	Higher	<0.0001
CM vs. VP	Proportion Dead	ns	Lower	0.1252
CM vs. ZP	Proportion Dead	****	Higher	<0.0001
KCS vs. KLS	Proportion Dead	****	Lower	<0.0001
KCS vs. M	Proportion Dead	ns	Higher	0.0587
KCS vs. VP	Proportion Dead	***	Lower	0.0002
KCS vs. ZP	Proportion Dead	*	Higher	0.0317
KLS vs. M	Proportion Dead	****	Higher	<0.0001
KLS vs. VP	Proportion Dead	ns	Higher	0.4632
KLS vs. ZP	Proportion Dead	****	Higher	<0.0001
M vs. VP	Proportion Dead	****	Lower	<0.0001
M vs. ZP	Proportion Dead	ns	Higher	>0.9999
VP vs. ZP	Proportion Dead	****	Higher	<0.0001
B vs. G	Proportion Viable	ns	Higher	0.8162
B vs. CM	Proportion Viable	****	Higher	<0.0001
B vs. KCS	Proportion Viable	****	Higher	<0.0001
B vs. KLS	Proportion Viable	****	Higher	<0.0001
B vs. M	Proportion Viable	**	Higher	0.0072
B vs. VP	Proportion Viable	****	Higher	<0.0001
B vs. ZP	Proportion Viable	*	Higher	0.0143
G vs. CM	Proportion Viable	****	Higher	<0.0001
G vs. KCS	Proportion Viable	****	Higher	<0.0001
G vs. KLS	Proportion Viable	****	Higher	<0.0001
G vs. M	Proportion Viable	ns	Higher	0.215
G vs. VP	Proportion Viable	****	Higher	<0.0001
G vs. ZP	Proportion Viable	ns	Higher	0.335
CM vs. KCS	Proportion Viable	ns	Higher	0.2319
CM vs. KLS	Proportion Viable	***	Higher	0.0007
CM vs. M	Proportion Viable	****	Lower	<0.0001
CM vs. VP	Proportion Viable	ns	Higher	0.1252
CM vs. ZP	Proportion Viable	****	Lower	<0.0001
KCS vs. KLS	Proportion Viable	****	Higher	<0.0001
KCS vs. M	Proportion Viable	ns	Lower	0.0587
KCS vs. VP	Proportion Viable	***	Higher	0.0002
KCS vs. ZP	Proportion Viable	*	Lower	0.0317
KLS vs. M	Proportion Viable	****	Lower	<0.0001
KLS vs. VP	Proportion Viable	ns	Lower	0.4632
KLS vs. ZP	Proportion Viable	****	Lower	<0.0001
M vs. VP	Proportion Viable	****	Higher	<0.0001
M vs. ZP	Proportion Viable	ns	Lower	>0.9999
VP vs. ZP	Proportion Viable	****	Lower	<0.0001

a ConvaMax™ Superabsorber (CS), 3M™ Kerramax™ Care Super-Absorbent Dressing (KCS), Kliniderm® Superabsorbent (KLS), Mextra® Superabsorbent (MS), Vliwasorb® Pro (VP), and Zetuvit® Plus (ZP).

b not significant (ns).

c significantly (or a trend towards) higher or lower proportion compared to the other dressing.

Supplementary Table 11: Significant differences for *P. aeruginosa* viability at day 7 (Figure 6c).

Comparison [a]	Type	Significance [b]	Higher/Lower [c]	P Value
B vs. G	Proportion Dead	****	Lower	<0.0001
B vs. CM	Proportion Dead	****	Lower	<0.0001
B vs. KCS	Proportion Dead	****	Lower	<0.0001
B vs. KLS	Proportion Dead	****	Lower	<0.0001
B vs. M	Proportion Dead	****	Lower	<0.0001
B vs. VP	Proportion Dead	****	Lower	<0.0001
B vs. ZP	Proportion Dead	ns	Lower	0.9945
G vs. CM	Proportion Dead	*	Lower	0.0278
G vs. KCS	Proportion Dead	ns	Lower	0.3113
G vs. KLS	Proportion Dead	****	Lower	<0.0001
G vs. M	Proportion Dead	ns	Higher	0.8457
G vs. VP	Proportion Dead	****	Lower	<0.0001
G vs. ZP	Proportion Dead	****	Higher	<0.0001
CM vs. KCS	Proportion Dead	ns	Higher	0.9377
CM vs. KLS	Proportion Dead	****	Lower	<0.0001
CM vs. M	Proportion Dead	***	Higher	0.0006
CM vs. VP	Proportion Dead	**	Lower	0.0016
CM vs. ZP	Proportion Dead	****	Higher	<0.0001
KCS vs. KLS	Proportion Dead	****	Lower	<0.0001
KCS vs. M	Proportion Dead	*	Higher	0.0148
KCS vs. VP	Proportion Dead	****	Lower	<0.0001
KCS vs. ZP	Proportion Dead	****	Higher	<0.0001
KLS vs. M	Proportion Dead	****	Higher	<0.0001
KLS vs. VP	Proportion Dead	ns	Higher	0.1935
KLS vs. ZP	Proportion Dead	****	Higher	<0.0001
M vs. VP	Proportion Dead	****	Lower	<0.0001
M vs. ZP	Proportion Dead	****	Higher	<0.0001
VP vs. ZP	Proportion Dead	****	Higher	<0.0001
B vs. G	Proportion Viable	****	Higher	<0.0001
B vs. CM	Proportion Viable	****	Higher	<0.0001
B vs. KCS	Proportion Viable	****	Higher	<0.0001
B vs. KLS	Proportion Viable	****	Higher	<0.0001
B vs. M	Proportion Viable	****	Higher	<0.0001
B vs. VP	Proportion Viable	****	Higher	<0.0001
B vs. ZP	Proportion Viable	ns	Higher	0.9945
G vs. CM	Proportion Viable	*	Higher	0.0278
G vs. KCS	Proportion Viable	ns	Higher	0.3113
G vs. KLS	Proportion Viable	****	Higher	<0.0001
G vs. M	Proportion Viable	ns	Lower	0.8457
G vs. VP	Proportion Viable	****	Higher	<0.0001
G vs. ZP	Proportion Viable	****	Lower	<0.0001
CM vs. KCS	Proportion Viable	ns	Lower	0.9377
CM vs. KLS	Proportion Viable	****	Higher	<0.0001
CM vs. M	Proportion Viable	***	Lower	0.0006
CM vs. VP	Proportion Viable	**	Higher	0.0016
CM vs. ZP	Proportion Viable	****	Lower	<0.0001
KCS vs. KLS	Proportion Viable	****	Higher	<0.0001
KCS vs. M	Proportion Viable	*	Lower	0.0148
KCS vs. VP	Proportion Viable	****	Lower	<0.0001
KCS vs. ZP	Proportion Viable	****	Lower	<0.0001
KLS vs. M	Proportion Viable	****	Lower	<0.0001
KLS vs. VP	Proportion Viable	ns	Lower	0.1935
KLS vs. ZP	Proportion Viable	****	Lower	<0.0001
M vs. VP	Proportion Viable	****	Higher	<0.0001
M vs. ZP	Proportion Viable	****	Lower	<0.0001
VP vs. ZP	Proportion Viable	****	Lower	<0.0001

a ConvaMax™ Superabsorber (CS), 3M™ Kerramax™ Care Super-Absorbent Dressing (KCS), Kliniderm® Superabsorbent (KLS), Mextra® Superabsorbent (MS), Vliwasorb® Pro (VP), and Zetuvit® Plus (ZP).

b not significant (ns).

c significantly (or a trend towards) higher or lower proportion compared to the other dressing.
